# Image restoration and key field alignment for misaligned overlapping text in secondary printing document images

**DOI:** 10.3389/frai.2025.1616007

**Published:** 2025-09-01

**Authors:** Senlong Wang, Junchao Ge, Jiantao Zhang, Hong He, Yunwei Zhang

**Affiliations:** ^1^Faculty of Information Engineering and Automation, Kunming University of Science and Technology, Kunming, China; ^2^Higher Educational Key Laboratory for Industrial Intelligence and Systems of Yunnan Province, Kunming, China

**Keywords:** secondary printed document images, text overlap, OCR recognition, image restoration, key-field alignment

## Abstract

With the advancement of information technology, the demand for efficient recognition and information extraction from paper documents in industrial scenarios has grown rapidly. In practice, business information is often secondarily printed onto pre-designed templates, which frequently leads to text misalignment or overlap with backgrounds and tables, thereby significantly impairing the accuracy of subsequent Optical Character Recognition (OCR). To address this issue, this paper proposes a preprocessing method for OCR recognition of secondary printed documents, specifically targeting the problems of text misalignment and overlap. In particular, we design a Text Overlap Restoration Network (TORNet) to restore document images affected by text overlap. Experimental results demonstrate that, compared to the latest image restoration models, TORNet achieves PSNR improvements of 0.17 dB and 0.12 dB in foreground and background text restoration, respectively. Furthermore, to resolve residual misalignment issues after image restoration, a key-field alignment method is introduced. This method accurately locates the positional deviations of critical fields in the reconstructed image, enabling precise field-level alignment and structural correction. Based on the proposed preprocessing framework, the recognition accuracy and field-matching accuracy are improved by 23% and 31%, respectively, compared to existing commercial OCR models, significantly enhancing the recognition performance on misaligned and overlapping documents. This study provides an effective solution for recognizing secondary printed documents with text overlap in industrial environments.

## 1 Introduction

With the advancement of information technology, the rapid and accurate recognition of printed paper documents has become increasingly important in industrial production. It is often necessary to automatically extract business-related information from these documents to improve workflow efficiency. In practice, a common approach involves secondary printed of business information onto pre-printed paper templates containing tables or form labels. However, due to various uncontrollable factors, this process can easily lead to misalignment or overlap between the newly printed text and the background text or table lines. This not only reduces the readability of the document but also severely hinders the accuracy of Optical Character Recognition (OCR) systems (Lalwani and Ramasamy, 2024). As a result, some enterprises still rely on manual data entry to avoid the recognition errors introduced by OCR under such conditions. Nevertheless, manual entry is inefficient, costly, and prone to human error, making it unsuitable for large-scale, automated business scenarios.

Traditional Optical Character Recognition (OCR) techniques have been widely applied in various text recognition tasks and have demonstrated satisfactory performance under standard conditions (Suzuki et al., 2003; Chen et al., 2022; Mei et al., 2021). However, their text detection and recognition pipelines often rely on predefined layout regions, making them less adaptable to complex layouts involving misaligned or overlapping characters. In practical documents where text frequently overlaps with table lines or exceeds predefined field boundaries, conventional OCR methods struggle to accurately detect and reconstruct the intended characters, and they are also limited in establishing semantic relationships and spatial alignment between fields.

**Figure 1:**
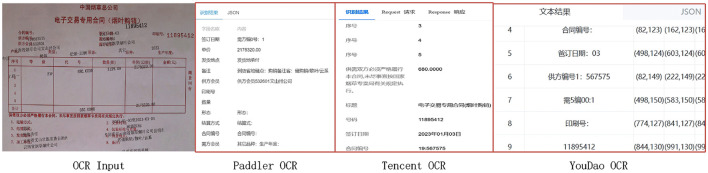
Different business OCR models recognizing text-overlapped and misaligned document images.

As illustrated in Figure 1, commercial OCR systems such as PaddleOCR, Tencent OCR, and Youdao OCR exhibit clear limitations when handling complex documents containing overlapping characters and misaligned fields. Specifically, these systems often suffer from recognition errors, failure to detect certain text regions, and inaccurate key-field matching. Such issues significantly hinder the reliability and effectiveness of automated document recognition in practical applications.

Although recent research on low-quality text recognition has achieved certain advancements, enhancing model robustness against issues such as blurred (Peng and Wang, 2020; Mou et al., 2020; Wang et al., 2021; Albahli et al., 2021), distorted (Zhu et al., 2020; Li et al., 2024; Zheng et al., 2024; Wu et al., 2022), incomplete characters (Niu et al., 2024; Villespin et al., 2024; Feng et al., 2023), and background noise (Wang et al., 2020; Yu et al., 2023; Yang et al., 2022; Ping et al., 2019), these methods typically assume that each input contains a single, isolated text target. As a result, they face limitations in handling scenarios involving overlapping characters or misaligned fields, leading to challenges in character separation and incomplete recognition. Furthermore, misaligned fields often extend beyond predefined detection boxes, further reducing the accuracy of field-level matching. Meanwhile, with the emergence of large-scale pre-trained models, their strong capabilities in semantic understanding and reasoning have shown promising adaptability in complex document recognition tasks (Abdellatif et al., 2025; Bourne, 2025). For images with overlapping text, such models can partially recover semantic content and infer field-level information. However, field misalignment continues to disrupt the logical structure and semantic coherence between fields, leading to a decline in final matching accuracy and thus limiting their effectiveness in fine-grained information extraction tasks.

In recent years, image preprocessing techniques leveraging deep neural networks have gained significant attention as a promising approach to enhance the recognition performance of complex document images. Unlike traditional image processing methods, deep learning models exhibit advanced capabilities in feature extraction and pattern recognition. These models can autonomously learn the structural distinctions between characters and backgrounds from large-scale datasets, without relying on manually engineered rules, thus providing more reliable and higher-quality image inputs for subsequent Optical Character Recognition (OCR) tasks (Lalwani and Ramasamy, 2024). Among these, Convolutional Neural Networks (CNNs) (Dong et al., 2014; Shanthakumari et al., 2022) have been widely adopted in tasks such as image denoising, enhancement, and super-resolution reconstruction, owing to their advantages in local perception and parameter sharing. However, convolutional operations inherently rely on fixed receptive fields and are limited in capturing long-range dependencies within an image. This becomes particularly problematic in scenarios involving cross-field overlaps or irregular spatial distribution of characters, where locally modeled features by CNNs often fail to recover complete semantic structures, thus constraining the effectiveness of image preprocessing. Recently, the Transformer architecture has offered a new paradigm for document image preprocessing tasks. By leveraging the self-attention mechanism, Transformers enable feature interactions across arbitrary positions in the image (Kim et al., 2022; Oubah and Ener, 2024; Zhou et al., 2024; Lou et al., 2025), thereby capturing global dependencies and modeling holistic semantic structures. Nonetheless, their ability to capture fine-grained local details remains limited.

To address the aforementioned challenges, this paper proposes a preprocessing method for OCR recognition of secondary printed documents, specifically targeting the issues of text misalignment and overlap. The aim is to systematically correct character superposition and positional dislocation that frequently occur during the secondary printing process in industrial documents. The proposed method comprises two core components: (1) structural restoration of overlapped text images, and (2) precise alignment and positional correction of key fields within the restored images. Through these preprocessing steps, document images with complex misalignment and overlap issues can be transformed into well-structured, clearly separated inputs suitable for standard OCR systems, thereby significantly improving the accuracy of subsequent text recognition and field matching. To meet the demands of restoring heavily overlapped regions, we design a novel image restoration model named Text Overlap Restoration Network (TORNet). TORNet integrates the strengths of Convolutional Neural Networks (CNNs) in modeling local details with the capabilities of Transformer architectures in capturing global structural relationships. This hybrid architecture enables joint modeling of both local perceptual features and global semantic structures, facilitating accurate restoration of characters affected by secondary printing overlaps. Furthermore, to enhance structural alignment in the recognition process, we propose a key field alignment method that detects and analyzes spatial deviations of important fields within the reconstructed image. This enables precise field-level localization and structural correction, effectively compensating for residual positional errors after image restoration. The proposed method significantly improves the field-level matching stability of OCR systems and enhances recognition performance for secondary printing documents in complex real-world scenarios.

Overall, the main contributions of this paper are summarized as follows:

- To address the common issues of text overlap and field misalignment in secondary printed documents within industrial scenarios, we propose a systematic preprocessing pipeline. This framework restores and aligns character structures from complex document images, providing a more reliable input foundation for downstream OCR recognition.
- The proposed Text Overlap Restoration Network (TORNet) integrates the local feature extraction strength of CNNs with the global context modeling ability of Transformers, enabling effective recovery of textual information in scenarios involving character-table overlaps and misalignments. TORNet demonstrates robust performance in restoring text under structurally complex document layouts.
- We propose a key-field alignment strategy. This method performs precise localization and offset correction of critical fields, achieving field-level structural alignment and significantly improving the accuracy and robustness of field matching.

## 2 Related work

### 2.1 Optical character recognition for secondary printed documents

In this section, we review the related work on OCR techniques for degraded or secondary printed documents. Several studies have explored innovative methods to enhance text extraction accuracy under challenging conditions such as noise, skew, and overlapping text. For instance, Zhao et al. (2020) proposed a robust OCR framework combining image preprocessing and deep learning to extract text from noisy documents, achieving significantly improved recognition accuracy. Similarly, Thorat et al. (2023) emphasized the importance of noise reduction and binarization in preprocessing, coupled with CNN and CRNN models, to effectively handle degraded inputs and improve OCR reliability. Lalwani and Ramasamy (2024) introduced a hybrid approach using CNNs and BiLSTMs, addressing both spatial and sequential features for handwritten and printed text extraction, which is especially relevant for secondary printing documents with alignment issues. Their model outperformed traditional techniques in terms of accuracy and adaptability. In another study, U et al. (2023) leveraged MSER algorithms for stable text region detection and combined them with CNN-based OCR, yielding better results on images with complex content and varied character sets.

Despite these advances, there remains a gap in OCR systems specifically optimized for the characteristics of secondary printed industrial documents, especially handling repeated stamps, layout inconsistencies, and visual noise. The present work addresses this gap by proposing an image restoration and key field alignment method tailored to the OCR processing of secondary printing industrial bills.

### 2.2 Image restoration

The problem of restoring overlapping and misaligned text in secondary printing industrial documents lies at the intersection of image restoration and OCR. Recent progress in deep learning has introduced various model types that significantly advance these tasks. This section categorizes the related work based on model architectures.

#### 2.2.1 CNN-based image restoration models

Convolutional Neural Networks (CNNs) have laid the foundation for most early developments in image restoration. Models such as SRCNN (Dong et al., 2014), DnCNN (Zhang et al., 2016), and ARCNN (Dong et al., 2015) initiated a wave of research focused on learning mappings from low-quality to high-quality images using large paired datasets. Subsequent works improved these architectures by introducing advanced components, including residual blocks (Kim et al., 2016; Zhang et al., 2022), dense blocks (Zhang et al., 2021), attention mechanisms (Zhang et al., 2018b; Mei et al., 2021; Niu et al., 2020), and skip connections, greatly enhancing feature representation and image restoration capabilities. Models like Shift-Net (Yan et al., 2018), which shifts encoder features into decoder space for semantic filling, and PEN-Net (Zeng et al., 2019), which captures multi-scale contextual semantics, exemplify the success of CNN-based encoder-decoder architectures such as U-Net in repairing irregular or defect-laden image regions. Multi-stage models such as MPRNet (Zamir et al., 2021) and DGUNet (Mou et al., 2022) further address the limitations of single-pass restoration by progressively refining outputs across stages and scales. Additionally, Feng et al. (2021) proposed DocScanner, a progressive learning-based framework for robust document image rectification. It achieves strong performance in handling complex distortions and irregular layouts, providing valuable insights for correcting secondary-printed document artifacts.

#### 2.2.2 Transformer-based models for image restoration

Inspired by the success of Transformers in NLP, researchers have begun to explore their applications in image restoration. AOT-GAN (Zeng et al., 2023) employs an aggregated context transformer for structural inpainting, while SwinIR (Liang et al., 2021) adapts the Swin Transformer to image restoration, balancing performance and model complexity. MAE (He et al., 2022), a masked autoencoder, enables self-supervised training for high-fidelity restoration, and Restormer (Zamir et al., 2022) combines MDTA and GDFN modules to efficiently model long-range pixel dependencies while maintaining high-resolution detail. Zhang et al. (2024b) proposed STUNet, a Swin Transformer-based U-Net for blind image restoration, demonstrating strong performance under complex and unknown degradations. Li et al. (2025) developed a memory-augmented Transformer for document stain removal, highlighting its effectiveness in handling background interference. Zhang et al. (2024a) introduced a dual-attention network combining convolution and Transformer modules, offering useful insights for degraded input restoration.

In the context of integrating large language models (LLMs) for OCR post-processing, recent studies have increasingly explored combining LLMs with OCR refinement to enhance text extraction and structural understanding in complex document scenarios. PreP-OCR proposes joint image restoration and LLM-based correction to improve OCR quality (Guan et al., 2025); DocLayLLM integrates visual patch and spatial embeddings into LLM input for joint modeling of text and layout (Liao et al., 2025); LapDoc introduces rule-based layout prompts to enhance structural perception of LLMs (Lamott et al., 2024); and LayTextLLM encodes bounding boxes as tokens interleaved with text to unify content and layout representation (Lu et al., 2024).

Despite advancements in both CNN- and Transformer-based restoration and OCR models, there remains a lack of specialized solutions for secondary printed industrial documents. These documents often contain repetitive stamps, visual degradation, and key-field dislocation, which are insufficiently handled by current approaches. Although large language models have shown strong performance in OCR correction and post-processing, they still struggle with complex cases such as character overlaps and misaligned field matching, often resulting in incomplete recognition or semantic misinterpretation. This study aims to address these challenges by proposing an integrated framework that combines document image restoration with key-field alignment, specifically designed to enhance OCR accuracy for industrial document digitization.

## 3 Methods

### 3.1 Algorithm flowchart

**Figure 2:**
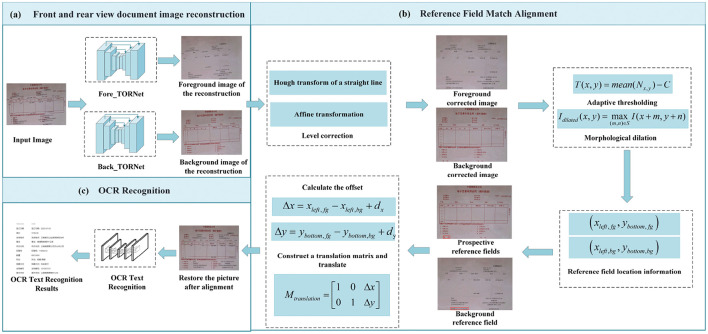
Workflow of the proposed algorithm.

The paper proposes an efficient preprocessing framework for OCR to address the common issues of character overlap and misalignment in industrial invoices during the secondary printing process, significantly improving the accuracy of subsequent text recognition. The method primarily consists of two stages: document image restoration and misaligned content correction with image fusion, as illustrated in Figure 2.

#### 3.1.1 Document image restoration

Industrial invoices often suffer from image degradation, such as character overlap and positional shifts, due to repeated printing, stamping, or multiple scans. These issues significantly hinder Optical Character Recognition (OCR) performance. To address this, we propose the use of a Text Overlap Restoration Network (TORNet) to recover the original document layout. This model reconstructs degraded images into two separate layers: a foreground image and a background image, both free from visual interference and redundant artifacts. The separation of these layers facilitates precise structural alignment in subsequent stages.

#### 3.1.2 Misaligned content correction and image fusion

Initially, Hough line detection is employed on both the foreground and background restoration images, succeeded by affine transformation to rectify horizontal misalignments. After geometric correction, adaptive thresholding and morphological dilation are applied to extract text regions within key reference fields and determine their spatial coordinates. Based on the positional relationships among these reference fields, a translation matrix is constructed to adjust the foreground image, ensuring accurate alignment with the background. Finally, the corrected foreground is pixel-wise fused with the background image to produce a clear document image free from character overlap and misalignment. This refined image is then passed to the OCR system, significantly enhancing recognition accuracy and stability.

### 3.2 Restoration of document images with overlapping text

This paper introduces an efficient Transformer-based architecture specifically designed for the restoration of text-overlapped document images. To overcome the limitations of multi-head self-attention (MHSA) in modeling local details and the insufficient global context representation of CNNs, we propose a hybrid mechanism that combines attention and convolution to enhance hierarchical feature extraction. Additionally, we design a multi-scale feedforward network to extract features across multiple resolutions, effectively reducing detail loss and structural ambiguity in overlapped regions, thereby improving restoration performance on complex document images.

#### 3.2.1 Overall pipeline

**Figure 3:**
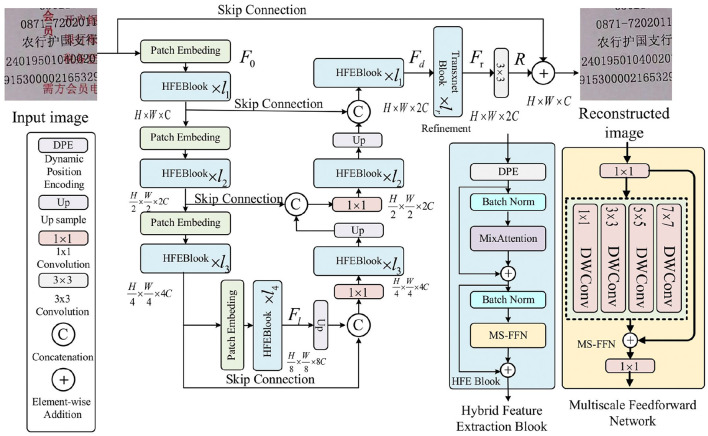
The proposed text overlap restoration network (TORNet) structure.

The network model proposed in this paper is shown in Figure 3. Given a low-quality image *I* ∈ ℝ^*H* × *W* × 3^, the model first constructs a Patch Embedding layer through a convolutional layer to divide the input image into small chunks and to obtain the low-level feature embedding $F_{0}\in {\mathbb{R}}^{H\times W\times C}$, where *H* × *W* represents the spatial dimensions and *C* is the number of channels. Next, the shallow features *F*_0_ are transformed into deep features $F_{d}\in {\mathbb{R}}^{H\times W\times 2C}$ via an encoder-decoder.

Each level of the encoder-decoder consists of multiple Hybrid Feature Extraction (HFE) blocks, which increase in number from shallow to deep to enhance efficiency. The encoder processes high-resolution inputs, reducing spatial dimensions and expanding channel depth, while the decoder gradually recovers high-resolution representations from low-resolution latent features $F_{l}\in {\mathbb{R}}^{H/8\times W/8\times 8C}$. Each HFE block combines a Dynamic Position Encoding (DPE) layer, a MixAttention mechanism, and a Multi-scale Feedforward Network (MS-FFN) to jointly model local and global information. The DPE layer adapts positional encoding based on the local and global context of the image, improving flexibility. The MixAttention mechanism merges the strengths of self-attention and convolution, enabling dynamic focus across multiple scales. Finally, the MS-FFN extracts features at various scales, addressing detail loss and structural complexity, especially in text overlap and distortion, improving the model's sensitivity and accuracy.

Patch Embedding and pixel-shuffle (Shi et al., 2016) are applied, respectively. To enhance the restoration effect, the encoder's features are fused with the decoder through skip connections, and the number of channels in all layers (except the topmost layer) is halved by a 1 × 1 convolution operation after skip-connections. At the topmost layer, the HFE block aggregates the low-level image features of the encoder with the high-level features of the decoder, preserving the fine structure and texture details of the restored image.

Subsequently, a refinement stage at high spatial resolution further enriches the deep features *F*_*d*_. Finally, a residual image *R* ∈ ℝ^*H* × *W* × 3^ is generated through a convolutional layer and added to the input image *I* to obtain the final restored image: Î = *I* + *R*.

#### 3.2.2 MixAttention mechanism

**Figure 4:**
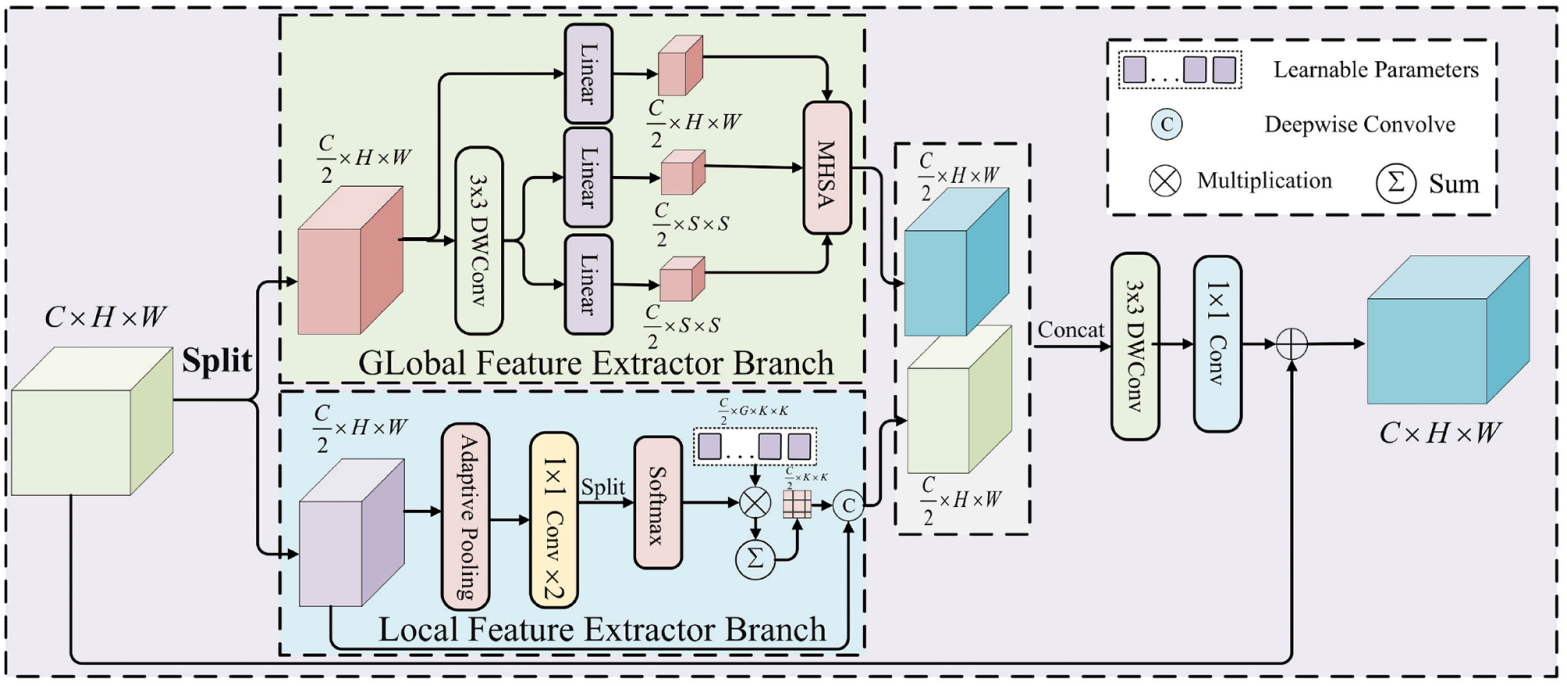
Architecture of the MixAttention mechanism, with a parallel dual-branch module for global and local feature extraction.

The MixAttention mechanism combines the advantages of global and local feature extraction, enabling it to capture both global and local information in images. Unlike traditional self-attention mechanisms, which focus only on global relationships, MixAttention dynamically adjusts the attention focus, allowing the model to adaptively extract multi-scale features from the image. Specifically, MixAttention designs a parallel dual-branch feature extraction module for global and local features, as shown in the Figure 4. which not only focuses on the overall structure of the image but also captures local details accurately. This structure enhances the model's adaptability to complex images, improving its feature extraction capability and making it more flexible and efficient in handling various visual tasks.

For the extracted feature map *X* ∈ ℝ^*H* × *W* × *C*^, it is first divided into two sub-feature maps $\left\{X_{1},X_{2}\right\}\in {\mathbb{R}}^{H\times W\times C/2}$, along the channel dimensions. These are then separately fed into the global feature extraction (GFE) module and the local feature extraction(LFE) module, generating the corresponding feature maps $\left\{X_{1}^{\prime },X_{2}^{\prime }\right\}\in {\mathbb{R}}^{H\times W\times C/2}$. Finally, the two extracted feature maps are aggregated by combining a 3 × 3 depthwise convolution, 1 × 1 channel squeeze-and-expansion convolutions, and a residual connection. The formula is as follows:


(1) $$\begin{array}{l} X_{1},X_{2}=\text{Split}\left(X\right) \end{array}$$

(2) $$\begin{array}{l} X^{\prime }=\text{Concat}\left(\text{GFE}\left(X_{1}\right),\text{LFE}\left(X_{2}\right)\right) \end{array}$$

(3) $$\begin{array}{l} Y={\text{Conv}}_{1\times 1}^{\left(C/r\to \left(G\times C\right)\right)}\left({\text{Conv}}_{1\times 1}^{\left(C\to C/r\right)}\left({\text{DWConv}}_{3\times 3}\left(X^{\prime }\right)\right)\right)+X \end{array}$$

Specifically, the Global Feature Extraction (GFE) module captures global information in the image by processing the input feature map *X*_1_, focusing on the overall structure and long-range contextual dependencies. This module effectively represents the spatial structure near the patch boundaries. This approach not only avoids the problems associated with reducing token counts using non-overlapping patches but also prevents the destruction of the spatial structure of patch boundaries, thereby reducing the degradation of token quality. To achieve this, the query matrix *Q* is first generated by applying a linear transformation to the input feature map *X*_1_:


(4) $$\begin{array}{l} Q=\text{Linear}\left(X_{1}\right) \end{array}$$

Next, the key and value matrices *K* and *V* are obtained by applying a linear transformation to the sum of a depthwise convolution (DWConv_3 × 3_) on *X*_1_ :


(5) $$\begin{array}{l} K,V=\text{Split}\left(\text{Linear}\left({\text{DWConv}}_{3\times 3}\left(X_{1}\right)+\text{LR}\left({\text{DWConv}}_{3\times 3}\left(X_{1}\right)\right)\right)\right) \end{array}$$

(6) $$\begin{array}{l} X_{1}=\text{Softmax}\left(\frac{QK^{T}}{\sqrt{d}}+B\right)V \end{array}$$

Where LR(·) denotes the local refinement module instantiated by a 3 × 3 depthwise convolution, B is the relative position bias matrix of the spatial relationships in the encoded attention map, and d is the number of channels for each attention head.

In contrast, the LFE module focuses on extracting fine-grained local information by processing the local regions of the input feature map *X*_2_, aiming to capture important features related to the image details. Given the input feature map $X_{2}\in {\mathbb{R}}^{H\times W\times C/2}$, adaptive average pooling is used to aggregate the spatial context, compressing the spatial dimensions to *K*^2^, and then forwarding it to two consecutive 1 × 1 convolutions to obtain the attention map *A*′ ∈ ℝ^(*G* × *C*/2) × *K*^2^^, where *G* denotes the number of attention groups. Next, *A*′ is reshaped into ℝ^(*G* × *C*/2) × *K*^2^^, and a Softmax function is applied along the *G*-dimension to generate the attention weights *A* ∈ ℝ^*G* × *C*/2 × *K*^2^^. Finally, *A* is multiplied element-wise by a set of learnable parameters *P* ∈ ℝ^*G* × *C*/2 × *K*^2^^, and the output is summed over the *G*-dimension to obtain the input-dependent deep convolution kernel *W* ∈ ℝ^*C*/2 × *K*^2^^. The deep convolution kernel *W* is convolved with the input feature map *X*_2_, capturing fine-grained feature details at multiple scales, thereby enhancing the feature representation capability. Specifically, the LFE operation can be expressed as:


(7) $$\begin{array}{l} A^{\prime }={\text{Conv}}_{1\times 1}^{\left(C/2r\to (G\times C/2\right)}\left({\text{Conv}}_{1\times 1}^{\left(C/2r\to (G\times C/2)\right)}\left(\text{AdaptivePool}(X_{2})\right)\right) \end{array}$$

(8) $$\begin{array}{l} A=\text{Softmax}\left(\text{Reshape}\left(A^{\prime }\right)\right) \end{array}$$

(9) $$\begin{array}{l} W=\overset{G}{\underset{i=0}{\sum }}P_{i}A_{i} \end{array}$$

(10) $$\begin{array}{l} X_{2}=W*X_{2} \end{array}$$

where In which, * denotes the convolution operation.

#### 3.2.3 Multi-scale feedforward network (MS-FFN)

Multi-scale feed-forward network (MS-FFN) uses four parallel deep convolutions of different scales to enhance the feature representation by cascading different scale convolutional layers with different feature information extracted; each convolution handles a quarter of the channels, which can efficiently capture the multi-scale information, and solves the problem that the number of channels in the implicit layer is larger, and the single-scaled token aggregation cannot be adequately represented;


(11) $$\begin{array}{l} \bar{X}=\sigma \left({\text{Conv}}_{1\times 1}\left(X\right)\right) \end{array}$$

(12) $$\begin{array}{l} \bar{X_{i}^{\prime }}=\text{Split}\left(\bar{X}\right) \end{array}$$

(13) $$\begin{array}{l} F_{si}={\text{Conv}}_{s_{i}\times s_{i}}\left(\bar{X_{i}^{\prime }}\right) \end{array}$$

(14) $$\begin{array}{l} F=\text{Concat}\left(F_{1},F_{3},\ldots ,F_{si}\right) \end{array}$$

(15) $$\begin{array}{l} \text{MS-FFN}\left(X\right)=\sigma \left({\text{Conv}}_{1\times 1}\left(F+\bar{X}\right)\right) \end{array}$$

Where σ(·) denotes the LeakyReLU activation function, *i* ∈ [1, 2, 3, 4], *s*_*i*_ represents the kernel size, *s*_*i*_ ∈ [1, 3, 5, 7], and *F*_*s*_*i*__ corresponds to the output of the convolution layer with the respective kernel size.

#### 3.2.4 Optimizer and loss function

The TORNet in this paper uses the Adam optimizer, which can adaptively match the learning rate for different parameters, effectively improving the network's convergence speed and speeding it up to the optimum. In the image denoising and restoration task, the Charbonnier loss function is generally used for training. The Charbonnier loss is a smooth L1 loss, which has better numerical stability for small errors, especially when the error is close to zero, and can avoid the problem of gradient explosion. At the same time, the Charbonnier loss function is insensitive to outliers, so it has good robustness when dealing with data containing noise or outliers.


(16) $$\begin{array}{l} L_{\text{Charbonnier}}\left(\text{pred},\text{target}\right)=\sqrt{{\left(\text{pred}-\text{target}\right)}^{2}+\epsilon^{2}} \end{array}$$

Where pred denotes the output predicted by the model, and target denotes the outcome in the model wanted to, i.e., the label.

### 3.3 Alignment and the fusion of the restored misaligned content from secondary printing

In the secondary printing of misaligned documents, the misalignment of field correspondences and form contents can cause accuracy problems for subsequent OCR recognition. Therefore, it is necessary to correct the recovered image and perform field matching alignment to ensure that the text information of the image does not overlap when the recovered foreground and background document images are image fused.

Firstly, the overlapping misaligned document images are fed into the trained text misalignment overlap restoration network respectively to obtain text images containing only foreground text information and text images containing only background information; Next, the text tilt angle in the image is detected using the Hough straight-line transform. The foreground and background text pictures are corrected horizontally by affine transform, respectively, and the corrected foreground and background pictures are obtained. Subsequently, adaptive thresholding and morphological expansion operations are used to extract the text regions of the reference fields in the Foreground and background images. Adaptive thresholding separates the text region by calculating the dynamic threshold of the local region with the equation:


(17) $$\begin{array}{l} T\left(x,y\right)=\text{mean}\left(N_{x,y}\right)-C \end{array}$$

Where, *N*_*x, y*_ is the neighborhood pixels around the current pixel (*x, y*), mean(*N*_*x, y*_) is the mean value of the neighborhood pixels, and *C* is a constant to adjust the threshold value. Based on this threshold *T*(*x, y*), a pixel is labeled as foreground if *I*(*x, y*) ≥ *T*(*x, y*), and background otherwise. The morphological expansion operation expands the foreground region by structuring elements to fill in gaps that may exist after thresholding, with the formula:


(18) $$\begin{array}{l} I_{\text{dilated}}\left(x,y\right)=\underset{\begin{array}{c} \left(m,n\right)\in S \end{array}}{\max}I\left(x+m,y+n\right) \end{array}$$

Where *I*(*x, y*) is the original image, *S* is a structuring element, usually a small rectangular or circular structuring element, and (*m, n*) is the displacement of the structuring element. The expansion operation expands the pixel values of the foreground region in the image into the neighborhood, thus filling the gaps in the text region and enhancing the connectivity of the text region in the foreground and background images. Through the above processing, the text regions of the reference fields in the front and back view images can be extracted.

Through the above process, the text regions corresponding to the reference fields in the foreground and background images can be extracted. Assume a Cartesian coordinate system with the origin at the top-left corner, where the *x*-axis increases from left to right and the *y*-axis decreases from top to bottom (i.e., negative direction). The position of each reference field is recorded accordingly.

Let *x*_left, fg_ and *y*_bottom, fg_ denote the left and bottom boundaries of the foreground reference field, with length *L*_fg_ and height *H*_fg_. Similarly, let *x*_left, bg_ and *y*_bottom, bg_ represent the corresponding boundaries of the background field, with length *L*_bg_ and height *H*_bg_. To avoid overlapping text during image fusion, the spatial offsets between the foreground and background reference fields are calculated with correction terms. The displacement in the *x* and *y* directions is given by:


(19) $$\begin{array}{l} \Delta x=x_{\text{left},\text{ fg}}-x_{\text{left},\text{ bg}}+d_{x} \end{array}$$

(20) $$\begin{array}{l} \Delta y=y_{\text{bottom},\text{ fg}}-y_{\text{bottom},\text{ bg}}+d_{y} \end{array}$$

Where *d*_*x*_, *d*_*y*_ are correction values used to maintain proper text spacing during fusion and to prevent overlapping of foreground and background images' text areas. The values of *d*_*x*_ and *d*_*y*_ depend on the spatial distribution of the reference text fields. If the text fields are horizontally aligned, and the background reference text field is on the left while the foreground reference field is on the right, the correction values are defined as:


(21) $$\begin{array}{l} d_{y}=0.1;\;d_{x}=0.1+L_{\text{bg}} \end{array}$$

Conversely, if the foreground field precedes the background field, the values are:


(22) $$\begin{array}{l} d_{y}=0.1;\;d_{x}=0.1-L_{\text{bg}} \end{array}$$

For vertically aligned fields, where the background field is located above the foreground field, the corrections are set as:


(23) $$\begin{array}{l} d_{x}=0.1;\;d_{y}=0.1-H_{\text{bg}} \end{array}$$

If the foreground field is above the background field, then:


(24) $$\begin{array}{l} d_{x}=0.1;\;d_{y}=0.1+H_{\text{fg}} \end{array}$$

According to these, the offset in the X and Y directions is obtained. Construct the translation matrix *M*_translation_.


(25) $$\begin{array}{l} M_{\text{translation}}=\left[\begin{array}{ccc} 1 & 0 & \Delta x\\ 0 & 1 & \Delta y \end{array}\right] \end{array}$$

The foreground text image is translated by affine transformation to adjust its reference field position to the position of the background reference field while retaining the appropriate spacing. Finally, the corrected foreground and background text images are fused at the pixel level to generate a document image with non-overlapping text and corrected misalignment. This process effectively solves the recognition errors caused by text misalignment and provides accurate and reliable input for subsequent OCR. See Supplementary Material Section 1, Algorithm 1 and Figure S1 for the detailed Image Correction and Key Field Alignment algorithm and flow diagram.

## 4 Experiment and analysis

### 4.1 Experiment dataset

**Figure 5:**
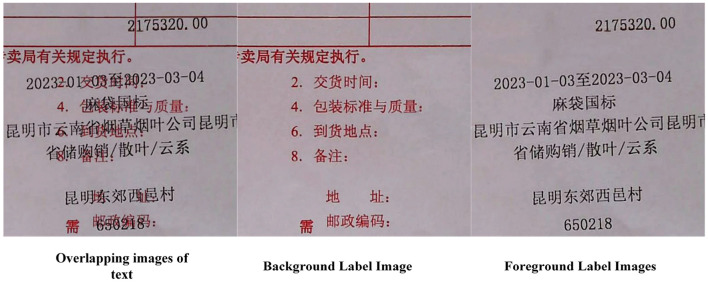
Schematic diagram of the dataset (model input, foreground label, background label).

In this study, a document image dataset was constructed specifically for the task of text overlap restoration. The dataset comprises 500 overlapping document images–including both real and synthetic samples–along with their corresponding foreground and background images. All three image types are precisely aligned in the pixel space, ensuring consistent annotation and high spatial registration accuracy. Real images were collected from actual printing scenarios, while synthetic images were generated by applying geometric transformations and image fusion techniques to simulate common text overlap patterns, thereby enhancing the diversity and coverage of the dataset. To improve training efficiency and the accuracy of detail restoration, all images were cropped into 128 × 128-pixel patches. Invalid samples were removed through a cleaning process, resulting in a total of 127,017 valid image patches. The dataset was subsequently divided into training, validation, and test sets in an 8:1:1 ratio, covering the three categories of data: overlapping images (as model inputs), foreground images (for foreground supervision), and background images (for background supervision). An illustration of the dataset structure is shown in Figure 5. See Supplementary Material Section 3, Figure S3 for detailed dataset production procedures, including model input, foreground label, and background label.

Based on this dataset, two subtasks were designed: foreground text restoration and background text restoration. Both subtasks take overlapping images as input, with the foreground or background images serving as supervision labels, respectively. These tasks are used to evaluate the model's ability to separate and reconstruct text content under conditions of visual overlap.

Although this study primarily focuses on the restoration of document images with text overlap, we further evaluate the generalization ability and robustness of the proposed model by applying it to a standard image denoising task involving uniformly distributed noise in color images. This auxiliary experiment serves two main purposes: (1) to demonstrate that the proposed architecture is not limited to document-specific degradations but is also effective for general-purpose image restoration; and (2) to showcase the model's capability in handling various noise types, including the complex and uneven noise patterns commonly encountered in secondary printing scenarios. To evaluate performance on the denoising task, we utilize the publicly available DFWB dataset for training, which comprises four sub-datasets: DIV2K (Agustsson and Timofte, 2017) (800 images), Flickr2K (Timofte et al., 2017) (2,650 images), BSD500 (Arbelaez et al., 2010) (400 images), and WED (Ma et al., 2016) (4,744 images). For testing, we adopt four widely used benchmark datasets: CBSD68 (Martin et al., 2001), Kodak24 (Franzen, 1999), McMaster (Zhang et al., 2011), and Urban100 (Huang et al., 2015). Specifically, CBSD68 contains 68 color images of varying sizes; Kodak24 consists of 24 uniformly sized images featuring people and landscapes; McMaster provides 18 natural scene images; and Urban100 includes 100 images focusing on urban architectural structures.

### 4.2 Experimental setup

All experiments are conducted using the PyTorch framework on a single 24GB NVIDIA GeForce RTX 4090 GPU. During model training, the depth of each layer of Hybrid Feature Extraction (HFE) Block is set to [3, 3, 9, 3], and the number of final image optimization blocks is 4. The feature dimensions of the coding and decoding phases are [48, 96, 192, 384], and the size of the Local Feature Extractor Branch (LFE) convolution kernel is uniformly [7, 7, 7, 7]. The different layers use the MixAttention mechanism with the number of attention heads set to [1, 2, 4, 8]. For training, the input training single document image size is 128 × 128, the optimizer uses Adam to minimize the loss function for parameter updating, and the optimizer parameters β_1_ and β_2_ are set to 0.9 and 0.999, respectively. The initial learning rate is set to 2 × 10^−4^.

### 4.3 Evaluation indicators

In the foreground and background restoration experiments for document images, we adopt Peak Signal-to-Noise Ratio (PSNR) and Structural Similarity Index Measure (SSIM) as evaluation metrics to assess the quality of image restoration.

To comprehensively evaluate the effectiveness of document image restoration and alignment, this study introduces two performance metrics grounded in OCR recognition results: Character Accuracy Rate (CAR) and Field Matching Accuracy Rate (FMAR). These indicators are designed to quantitatively assess the precision of OCR outputs, particularly with respect to the recognition and localization of critical textual fields following alignment.

The Character Accuracy Rate (CAR) measures the proportion of correctly recognized characters by the OCR system, The calculation formula is as follows:


(26) $$\begin{array}{l} CAR=\frac{C_{\text{correct}}}{C_{\text{total}}}\times 100\% \end{array}$$

where *C*_correct_ denotes the number of correctly recognized characters, and *C*_total_ represents the total number of characters.

Field Matching Accuracy Rate (FMAR) evaluates the structural accuracy of key field recognition and alignment. It is calculated as:


(27) $$\begin{array}{l} \text{FMAR}=\frac{\text{Number of correctly matched fields}}{\text{Total number of fields}}\times 100\% \end{array}$$

FMAR is particularly critical in application scenarios where structured data extraction is required, such as in industrial document processing involving batch numbers, dates, brands, and quantities. A higher FMAR indicates that the aligned document exhibits clearer layout structures and more reliable textual segmentation, facilitating both accurate OCR recognition and dependable downstream data analytics. Conversely, a lower FMAR implies the presence of residual misalignment or field confusion, which may hinder effective information retrieval.

### 4.4 Image restoration results

Table 1 presents the performance of each model in reconstructing and restoring tobacco document images with texts overlapping two different colors. To validate the proposed models' effectiveness, we compare them with the classical restoration models DnCNN (Zhang et al., 2016), RRDB (Ma et al., 2020), DPIR (Zhang et al., 2022), SwinIR (Liang et al., 2021), Restormer (Zamir et al., 2022), and STUGNet (Zhang et al., 2024b).

**Table 1:** Quantitative comparison of foreground and background image restoration performance.

**Model**	**Params (M)**	**FLOPs (G)**	**Foreground**		**Background**	
			**PSNR**	**SSIM**	**PSNR**	**SSIM**
DnCNN	0.671	11.01	35.81	0.973	35.92	0.975
RRDB	16.624	252.00	35.93	0.974	36.12	0.978
DPIR	32.640	35.893	35.98	0.976	36.29	0.979
SwinIR	11.504	197.00	36.06	0.977	36.31	0.981
Restormer	26.112	38.721	36.13	0.978	36.32	0.981
STUNet	17.79	34.27	36.21	0.978	36.31	0.982
**TORNet (Ours)**	17.342	30.777	**36.38**	**0.979**	**36.43**	**0.982**

Bold values indicate the best performance for each column.

As shown in Table 1, the proposed TORNet exhibits superior performance in reconstructing foreground text in secondary-printed documents. Specifically, it outperforms SwinIR and Restormer by **0.32 dB** and **0.25 dB** in PSNR, respectively, and shows a PSNR improvement of **0.40–0.57 dB** compared to DnCNN, RRDB, and DPIR. It also surpasses STUNet by **0.17 dB** in PSNR and achieves a slightly better SSIM.

For background text restoration, TORNet also achieves competitive results, surpassing SwinIR (Liang et al., 2021) and Restormer (Zamir et al., 2022) by **0.12 dB** and **0.11 dB**, respectively, and outperforming DnCNN, RRDB, DPIR, and STUNet by margins ranging from **0.12 dB** to **0.51 dB**.

In terms of model complexity, under an input resolution of 128 × 128, TORNet strikes a favorable balance between parameter count (Parameters/M) and computational cost (FLOPs/G), while achieving the highest PSNR performance across all evaluated models.

**Figure 6:**
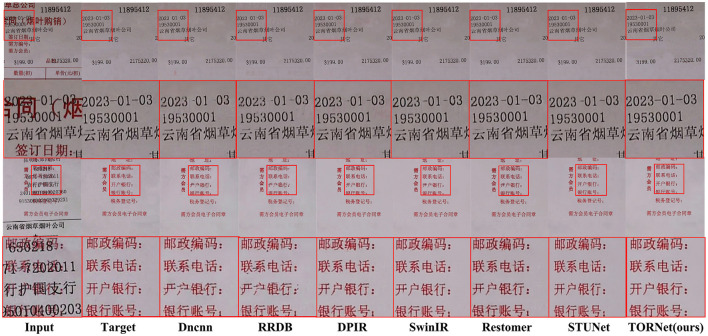
Visual comparison of foreground and background restoration results.

Figure 6 shows the visual effect of restoring the foreground and background information image of a tobacco document. It can be seen that DnCNN (Zhang et al., 2016) and RRDB (Ma et al., 2020) models show more obvious text loss during denoising; the other models, including STUNet, perform slightly better, though some shadowed or sticky artifacts remain.

### 4.5 Gaussian color image denoising results

**Figure 7:**
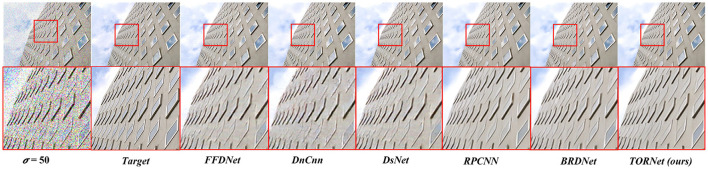
Color image denoising results at σ = 50.

Table 2 demonstrates the results of color image denoising. To verify the effectiveness of the proposed method in this paper, we compared the proposed model with several denoising models [DnCNN (Zhang et al., 2016), FFDNet (Zhang et al., 2018a), DSNet (Peng et al., 2019), RPCNN (Xia and Chakrabarti, 2020), and BRDNet (Tian et al., 2020)] at noise levels of 15, 25 and 50. As can be seen from the data in the table, the model in this paper exhibits superior image-denoising results in most PSNR evaluation metrics. In particular, on the Urban100 (Huang et al., 2015) dataset, the PSNR is improved by 0.83 dB, 0.82 dB, and 0.66 dB at noise levels of 15, 25, and 50, respectively, compared with the BRDNet (Tian et al., 2020) model. Figure 7 illustrates the color image denoising results for noise level σ = 50. The figure shows that the first three models still have noise after denoising and unsharp corners in the edge part. Despite the improvement of RPCNN (Xia and Chakrabarti, 2020) and BRDNet (Tian et al., 2020) the edges of the windows of the distant buildings are still blurred. The method proposed in this paper successfully avoids these problems, and the denoising effect is significantly better than other models.

**Table 2:** Quantitative comparison (average PSNR) with different color image denoising methods on a benchmark dataset.

**Method**	**CBSD68 (Martin et al., 2001)**			**Kodak24 (Franzen, 1999)**			**McMaster (Zhang et al., 2011)**			**Urban100 (Huang et al., 2015)**		
	σ = 15	σ = 25	σ = 50	σ = 15	σ = 25	σ = 50	σ = 15	σ = 25	σ = 50	σ = 15	σ = 25	σ = 50
FFDNet	33.87	31.21	27.96	34.63	32.13	28.98	34.66	32.35	29.18	33.87	31.21	27.96
DnCNN	33.90	31.24	27.95	34.60	32.16	29.05	33.45	31.52	28.62	33.90	31.24	27.95
DSNet	33.91	31.28	28.05	34.63	32.16	29.05	34.67	32.40	29.28	33.91	31.28	28.05
RPCNN	-	31.28	28.05	34.63	32.13	28.98	34.66	32.35	29.18	-	31.28	28.05
BRDNet	34.10	31.43	28.16	34.88	32.41	29.22	35.08	32.75	29.52	34.10	31.43	28.16
**TORNet (Ours)**	**34.29**	**31.60**	**28.42**	**35.16**	**32.6/5**	**29.58**	**35.39**	**33.03**	**29.98**	**34.93**	**32.25**	**28.80**

Bold values indicate the best performance for each column.

### 4.6 OCR recognition results

Table 3 presents the quantitative evaluation of OCR performance across different preprocessing stages using three mainstream OCR engines: PaddleOCR, Tencent OCR, and Youdao OCR. The evaluation is conducted on a dataset of 100 document images featuring various degrees of character misalignment and overlap, thereby ensuring a comprehensive and realistic benchmark for OCR under challenging conditions. Three progressive configurations are assessed: (1) no preprocessing, (2) image restoration only (TORNet), and (3) combined image restoration and text field alignment fusion (TFAF). In the baseline scenario without any preprocessing (Test IDs 1-3), recognition accuracy remains relatively low, ranging from 67% to 69%, while field match accuracy fluctuates between 58% and 60%. This outcome suggests that severe character overlap and misalignment in the raw images substantially hinder OCR performance. When image restoration is applied independently (Test IDs 4-6), both metrics improve significantly. Recognition accuracy increases to 81%–83%, and field match accuracy rises to 73%–74%, indicating that enhanced visual clarity facilitates more accurate character identification. The most substantial performance gains are observed when both image restoration and field alignment fusion are applied (Test IDs 7-9). PaddleOCR, for example, achieves 93% recognition accuracy and 94% field match accuracy. Tencent OCR and Youdao OCR exhibit comparable improvements, reaching 93%/92% and 92%/91%, respectively. These results highlight the complementary benefits of field-level semantic structuring in further boosting recognition consistency and precision. Overall, the proposed multi-stage preprocessing pipeline consistently improves OCR performance across all tested engines, demonstrating strong generalizability and effectiveness in enhancing both low-level text recognition and high-level field-level extraction accuracy.

**Table 3:** Quantitative evaluation of OCR performance improvements across progressive preprocessing stages.

**Test ID**	**TORNet**	**TFAF**	**OCR API**	**CAR (%)**	**FMAR (%)**
1	×	×	Paddle OCR	69	59
2	×	×	Tencent OCR	68	60
3	×	×	Youdao OCR	67	58
4	✓	×	Paddle OCR	81	74
5	✓	×	Tencent OCR	81	73
6	✓	×	Youdao OCR	83	74
7	✓	✓	Paddle OCR	93	94
8	✓	✓	Tencent OCR	93	92
9	✓	✓	Youdao OCR	92	91

**Figure 8:**
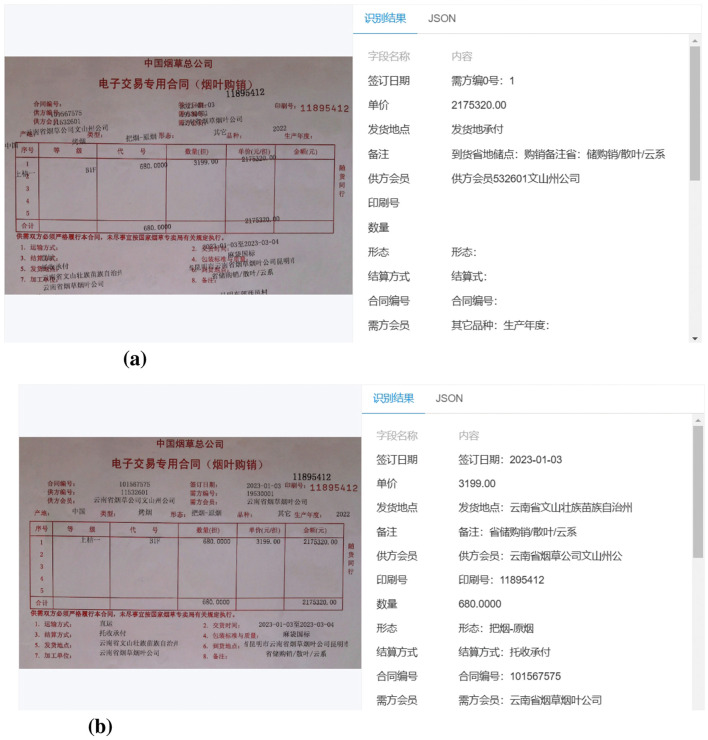
Visual results of OCR recognition before and after preprocessing. **(a)** OCR recognition on raw input image (PaddleOCR). **(b)** OCR recognition after preprocessing pipeline (PaddleOCR).

Figure 8a illustrates the OCR recognition results without applying any preprocessing, while Figure 8b presents sample results after employing the proposed method. As observed, the unprocessed images lead to chaotic OCR outputs, with frequent character recognition errors and misaligned field matching, making accurate information extraction difficult. In contrast, the images processed by our method exhibit clear improvements, with correctly recognized characters and accurately matched key fields, demonstrating the effectiveness of the proposed preprocessing pipeline. See Supplementary Material Section 2, Figure S2 and Table S1 for a more intuitive comparison of text recognition results and benchmark model performance.

### 4.7 Ablation experiments

#### 4.7.1 Effectiveness of individual components in TORNet

Table 4 presents the quantitative results of foreground and background image restoration using different variants of the proposed TORNet. The analysis focuses on evaluating the contribution of each individual component to the overall performance. The baseline model employs a plain U-shaped Transformer as the backbone, serving as a reference for subsequent module comparisons.

**Table 4:** Quantitative comparison of different methods on foreground and background image restoration.

**Method**	**Foreground image**		**Background image**	
	**PSNR**	**SSIM**	**PSNR**	**SSIM**
BaseLine	35.26	0.967	35.45	0.971
BaseLine + MS-FNN	35.63	0.971	35.74	0.975
BaseLine + MixAttention	36.14	0.976	36.31	0.979
**TORNet (Ours)**	**36.38**	**0.979**	**36.43**	**0.983**

Bold values indicate the best performance for each column.

##### 4.7.1.1 Effect of multi-scale feature fusion network (MS-FNN)

Introducing the MS-FNN module enables the model to effectively capture and integrate multi-resolution features. This results in a significant improvement in restoration performance. Compared to the baseline, MS-FNN enhances the model's capacity to recover fine-grained foreground structures and spatial background consistency.

##### 4.7.1.2 Effect of mixed attention mechanism (MixAttention)

Integrating the MixAttention module further boosts the model's representational capacity by jointly modeling local detail and global context. With MixAttention alone, the model achieves a foreground PSNR of 36.14 dB and SSIM of 0.976, while the background PSNR and SSIM reach 36.31 dB and 0.979, respectively. These results highlight the role of diverse attention mechanisms in improving restoration fidelity.

Furthermore, When both MS-FNN and MixAttention are combined, the complete TORNet model achieves the best performance across all metrics, with a foreground PSNR of 36.38 dB and SSIM of 0.979, and a background PSNR of 36.43 dB and SSIM of 0.983. These results demonstrate the complementary nature of the two modules and validate their joint effectiveness in enhancing restoration quality.

**Figure 9:**
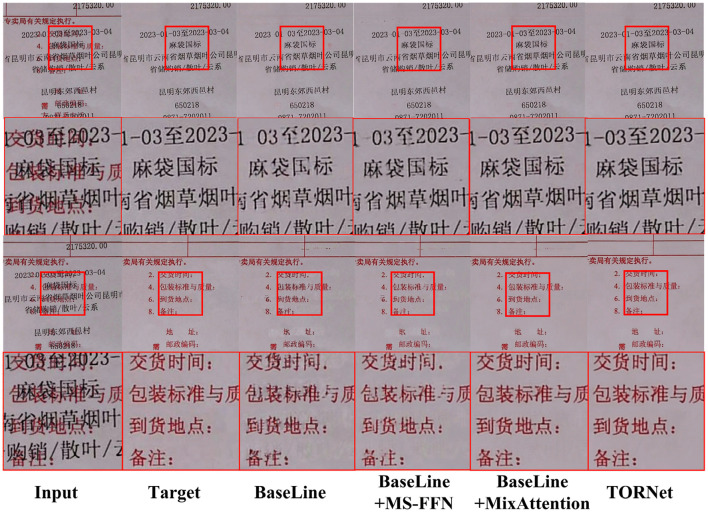
Comparison chart of ablation experiments.

Furthermore, Figure 9 presents the restoration results of different methods on typical samples. It can be observed that while the baseline model can restore the general contour, it suffers from significant blurring in the details and edges. After introducing MS-FNN, the local structures of the image are clearer, and the edge transitions become more natural. The combination with MixAttention further improves texture restoration and noise suppression. Ultimately, the TORNet restoration results visually align closely with the original images, highlighting the significant advantages of the proposed network.

#### 4.7.2 Downsampling schemes for document image restoration

The selection of downsampling strategies is crucial in balancing a model's parameter complexity, computational cost, and restoration quality. To systematically evaluate the impact of different downsampling modules on document foreground restoration, we compare two commonly employed techniques: *Patch Embedding* and *Pixel-Unshuffle*. With all other architectural components held constant, only the downsampling module is varied. We assess each configuration based on the number of parameters, floating-point operations (FLOPs), and peak signal-to-noise ratio (PSNR). As presented in Table 5, the Pixel-Unshuffle method results in a reduction of ~ 0.655 M parameters relative to Patch Embedding, making it a more lightweight option for scenarios with stringent model size constraints. However, the computational cost shows negligible difference between the two methods. In terms of restoration quality, Patch Embedding yields a marginally higher PSNR (36.38 vs. 36.23), suggesting that its patch-based representation facilitates richer feature extraction during downsampling, thereby enhancing document restoration performance.

**Table 5:** Comparison of downsampling methods for foreground restoration.

**Downsampling method**	**Parameters (M)**	**FLOPs (G)**	**PSNR**
Pixel-unshuffle	16.687	30.777	36.23
Patch-embedding	17.342	30.778	**36.38**

Bold values indicate the best performance for each column.

#### 4.7.3 Effect of initial patch embedding kernel size

To investigate the effect of kernel size in the initial Patch Embedding layer, we compare multiple configurations—3 × 3, 5 × 5, 7 × 7, and 9 × 9—while keeping all other components of the model unchanged. The quantitative results are presented in Table 6. Notably, the kernel size used in the initial convolutional projection directly determines the **patch size** fed into the Transformer. Therefore, these experiments essentially evaluate the impact of different patch sizes (i.e., spatial granularity) on the final performance. Smaller kernels correspond to finer patch division, allowing the model to focus on local texture variations, whereas larger kernels aggregate wider context in each patch. As shown in the table, increasing the kernel size from 3 × 3 to 7 × 7 yields a PSNR gain of **0.16 dB**, with negligible increases in parameter count and computational cost. Introducing the 5 × 5 kernel results in an intermediate improvement of 0.07 dB over the baseline, while the 9 × 9 configuration slightly underperforms compared to 7 × 7, indicating a potential saturation or decline beyond a certain receptive field size. This improvement suggests that a larger kernel facilitates more effective global context capture at the early feature extraction stage, thereby enhancing the quality of image reconstruction. However, excessively large kernels such as 9 × 9 may introduce redundant context or over-smooth local patterns, leading to marginal degradation. These findings underscore the importance of initial receptive field size (i.e., patch size) in tasks involving complex spatial patterns such as overlapping text restoration.

**Table 6:** Effect of initial patch embedding kernel size on foreground restoration performance.

**Kernel size**	**Params (M)**	**FLOPs (G)**	**PSNR**
3 × 3	17.336	30.684	36.22
5 × 5	17.338	30.721	36.29
7 × 7	17.342	30.778	**36.38**
9 × 9	17.347	30.854	36.36

Bold values indicate the best performance for each column.

#### 4.7.4 Dimensional changes on model performance

Table 7 compares different feature dimension configurations in terms of reconstruction performance and computational cost. The configuration [48, 96, 224, 448], despite having higher model complexity with 22.565 M parameters and 34.037G FLOPs, yields a lower PSNR of 35.93 dB. In contrast, the configuration [48, 96, 192, 384] achieves a superior PSNR of 36.38 dB while maintaining a more compact model with only 17.342 M parameters and 30.778G FLOPs. These results indicate that increasing feature dimensions beyond a certain threshold may introduce redundancy, leading to diminished performance. The lower-dimensional configuration not only provides better reconstruction of fine image details but also exhibits enhanced suppression of background interference. Conversely, the higher-dimensional model tends to generate more visual artifacts and text degradation, further confirming the trade-off between model complexity and effective feature representation.

**Table 7:** Effect of feature dimension configuration on restoration quality.

**Dimensions**	**Parameters (M)**	**FLOPs (G)**	**PSNR**
[48, 96, 224, 448]	22.565	34.037	35.93
[48, 96, 192, 384]	**17.342**	**30.778**	**36.38**

Bold values indicate the best performance for each column.

#### 4.7.5 Inference speed evaluation under varying input resolutions

To evaluate the practical applicability of the proposed document restoration and alignment system, we assessed its end-to-end processing efficiency under varying input resolutions. All experiments were conducted using PyTorch 1.13 on a single NVIDIA GeForce RTX 4090 GPU with 24 GB of memory.

The complete pipeline comprises two primary stages: (1) document image restoration, and (2) key-field matching with alignment and fusion. We measured the average runtime (in seconds) for each stage using two representative input sizes (128 × 128 and 512 × 512), and the results are summarized in Table 8. The impact of different input sizes on model performance is provided in Supplementary Material Section 3.1 and Table S2.

**Table 8:** Average runtime per sample (in seconds) under different input resolutions.

**Image size**	**Restoration time (s)**	**Alignment time (s)**	**Total time (s)**
128 × 128	0.13	0.012	0.14
512 × 512	1.18	0.038	1.22

The results demonstrate that our system achieves fast inference on low-resolution inputs, with a total average processing time of only 0.14 s per sample. For high-resolution inputs (512 × 512), the processing time increases to ~1.22 s, which remains acceptable for practical deployment in industrial scenarios. These findings confirm that the proposed method effectively balances restoration quality and computational efficiency.

It is worth noting that actual runtime performance may vary depending on hardware specifications and input resolution. Therefore, system parameters can be flexibly adjusted to accommodate specific deployment requirements.

## 5 Conclusion

This paper presents a preprocessing framework for addressing text misalignment and overlap issues in secondary-printed documents, aiming to enhance OCR performance. The proposed method consists of two main components: (1) restoration of document images with overlapping and misaligned text, and (2) position alignment of content after restoration. To tackle the image restoration problem caused by secondary printing, we propose a specialized network named TORNet (Text Overlap Restoration Network). TORNet is designed to extract and restore structural and textual features in degraded industrial document images. Experimental results demonstrate that TORNet outperforms existing methods on industrial document datasets, achieving notable improvements in PSNR and qualitative performance. Furthermore, the proposed approach effectively separates overlapping textual information and reconstructs clear, standard document images with minimal misalignment. By correcting and aligning dislocated content through image processing techniques, the method significantly improves OCR accuracy in both character recognition and field-level matching. It addresses critical challenges arising from overlapping and misaligned text, providing a practical solution for robust OCR in complex real-world scenarios.

## 6 Discussion

### 6.1 Comparison with commercial OCR solutions and industrial deployment outlook

Our proposed TORNet framework focuses specifically on restoring and aligning overlapped and misaligned text in secondary printed industrial documents, which distinguishes it from many commercial OCR engines. While commercial systems such as PaddleOCR, Tencent OCR, and Youdao OCR offer robust text recognition, they often underperform when faced with severely degraded or overlapped inputs without prior image enhancement. TORNet integrates advanced image restoration with key-field alignment to convert complex document images into cleaner OCR inputs, significantly improving recognition accuracy as demonstrated in our experiments. Regarding industrial deployment, the method has been successfully integrated into backend processing pipelines of tobacco industry document digitization systems, proving its practical viability. See Supplementary Material Section 3.2, Figures S4–S8 for practical deployment cases in industrial systems. Nonetheless, real-time performance and robustness under varying acquisition conditions remain to be improved. Future work will focus on enhancing efficiency and expanding adaptability to diverse industrial document types and scenarios.

### 6.2 Limitations and future work

Despite the promising results, several limitations exist. First, the current implementation does not fully meet real-time processing requirements for high-throughput industrial environments. Second, the restoration accuracy is affected by external factors such as variable lighting conditions and non-ideal camera angles; in particular, colored lighting (e.g., red or blue) can degrade character visibility and feature extraction. Third, while our model performs well on industrial printed documents, its generalization to other document types (e.g., handwritten forms, multilingual documents) requires further investigation and potential adaptation. Addressing these challenges will be the focus of our future research to improve the model's robustness, efficiency, and generalizability.

## Data Availability

The raw data supporting the conclusions of this article will be made available by the authors, without undue reservation.
