# Supplementary material for: Image restoration and key field alignment for misaligned overlapping text in secondary printing document images
Source: Front Artif Intell. 2025 Sep 1;8:1616007. doi: 10.3389/frai.2025.1616007 (PMC12434626; doi:10.3389/frai.2025.1616007)
Supplement: Supplementary file 1 [file Data_Sheet_1.pdf]

# *Supplementary Material*

## 1 ALIGNMENT AND THE FUSION OF THE RESTORED MISALIGNED CONTENT FROM SECONDARY PRINTING

**Algorithm 1** Image Correction and Field Alignment algorithm

**Require:** $I_{\text{overlap}}$ ▷ Input misaligned and overlapped document image
**Ensure:** $I_{\text{fused}}$ ▷ A fused document image with no character overlap or field matching errors.

**Step 1: Foreground And Background Image Restoration**
$I_{\text{fg}} \leftarrow \text{Fore\_TORNet}(I_{\text{overlap}}); \quad I_{\text{bg}} \leftarrow \text{Back\_TORNet}(I_{\text{overlap}})$
**Step 2: Skew Correction via Hough Transform and Affine Transformation**
$angle_{\text{fg}} \leftarrow \text{HoughTransform}(I_{\text{fg}}); \quad angle_{\text{bg}} \leftarrow \text{HoughTransform}(I_{\text{bg}})$
$I_{\text{fg}}^{\text{corr}} \leftarrow \text{AffineRotate}(I_{\text{fg}}, angle_{\text{fg}}); \quad I_{\text{bg}}^{\text{corr}} \leftarrow \text{AffineRotate}(I_{\text{bg}}, angle_{\text{bg}})$
**Step 3: Extraction of Reference Text Fields Regions**
$T_{\text{fg}} \leftarrow \text{AdaptiveThreshold}(I_{\text{fg}}^{\text{corr}}); \quad T_{\text{bg}} \leftarrow \text{AdaptiveThreshold}(I_{\text{bg}}^{\text{corr}})$
$R_{\text{fg}} \leftarrow \text{MorphologicalDilation}(T_{\text{fg}}); \quad R_{\text{bg}} \leftarrow \text{MorphologicalDilation}(T_{\text{bg}})$
$(x_{\text{left,fg}}, y_{\text{bottom,fg}}, L_{\text{fg}}, H_{\text{fg}}) \leftarrow \text{ExtractRefField}(R_{\text{fg}})$
$(x_{\text{left,bg}}, y_{\text{bottom,bg}}, L_{\text{bg}}, H_{\text{bg}}) \leftarrow \text{ExtractRefField}(R_{\text{bg}})$
**Step 4: Calculates correction offsets based on reference text field layout rules**
**if** Reference text field layout is horizontal **then**
  **if** FG is on right of BG **then**
    $d_y \leftarrow 0.1; \quad d_x \leftarrow 0.1 + L_{\text{bg}}$
  **else** ▷ FG is on left of BG
    $d_y \leftarrow 0.1; \quad d_x \leftarrow 0.1 - L_{\text{fg}}$
  **end if**
**else** ▷ Reference text field layout is vertical
  **if** FG is below BG **then**
    $d_x \leftarrow 0.1; \quad d_y \leftarrow 0.1 - H_{\text{bg}}$
  **else** ▷ BG is below FG
    $d_x \leftarrow 0.1; \quad d_y \leftarrow 0.1 + H_{\text{fg}}$
  **end if**
**end if**
Compute offsets: $\Delta x = x_{\text{left,fg}} - x_{\text{left,bg}} + d_x; \quad \Delta y = y_{\text{bottom,fg}} - y_{\text{bottom,bg}} + d_y$
**Step 5: Foreground Translation and Image Fusion**
Translation matrix:

$$M_{\text{translation}} = \begin{bmatrix} 1 & 0 & \Delta x \\ 0 & 1 & \Delta y \end{bmatrix}$$

$I_{\text{fg}}^{\text{aligned}} \leftarrow \text{AffineTranslate}(I_{\text{fg}}^{\text{corr}}, M_{\text{translation}})$
$I_{\text{fused}} \leftarrow \text{PixelwiseFusion}(I_{\text{fg}}^{\text{aligned}}, I_{\text{bg}}^{\text{corr}})$
**return** $I_{\text{fused}}$

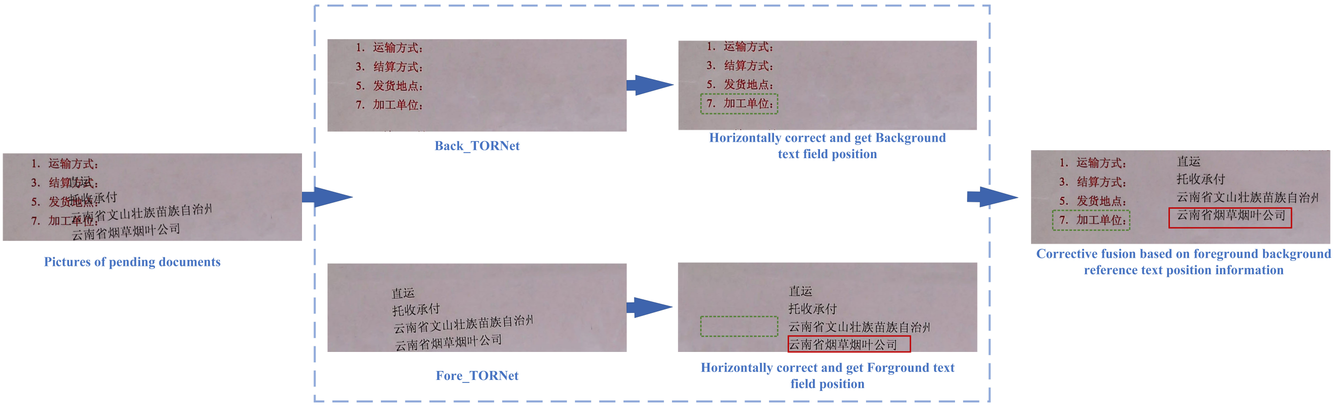


**Figure S1.** Flow diagram of key field alignment for Misaligned overlapping text

To better illustrate the field alignment and fusion process proposed in this paper, this chapter formalizes the specific steps into pseudocode, as shown in Algorithm 1 and Figure S1. The algorithm first describes how to separate foreground and background text from input overlapped and misaligned document images. It then corrects structural distortions in the document through geometric calibration and image alignment techniques. Next, adaptive thresholding and morphological operations are applied to extract text regions, further refining the candidate fields. Finally, these fields are matched to a predefined template through semantic similarity and positional alignment, ensuring that the final output contains accurate and structured document information

## 2 FIELD MATCHING RESULTS ON OVERLAPPED AND MISALIGNED DOCUMENT IMAGES

To overlap the more serious case of an overlapping misplaced part of a single document picture (shown in FigureS2) as an example, the use of Paddle IOCR, Deepseek_R1 inference model as well as ChatGPT_4o, as well as with the method proposed in this paper to the field identification matching effect show that the experimental data as shown in Table S1;

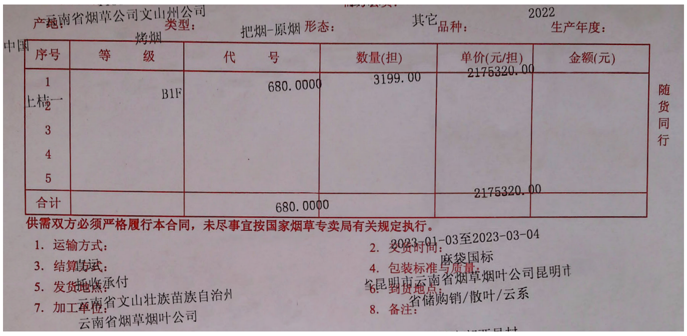
产地： 云南省烟草公司文山州公司 类型： 烤烟 把烟-原烟 形态： 其它 品种： 2022 生产年度：
中国

| 序号 | 等 级 | 代 号 | 数量(担) | 单价(元/担) | 金额(元) |
|---|---|---|---|---|---|
| 1 | B1F | 680.0000 | 3199.00 | 2175320.00 | |
| 2 | | | | | |
| 3 | | | | | |
| 4 | | | | | |
| 5 | | | | | |
| 合计 | | 680.0000 | | 2175320.00 | |

上桔一

随货同行

供需双方必须严格履行本合同，未尽事宜按国家烟草专卖局有关规定执行。

1. 运输方式： 直运
2. 交货时间： 2023-01-03至2023-03-04
3. 结算方式： 托收承付
4. 包装标准与质量： 麻袋国标
5. 发货地点： 云南省文山壮族苗族自治州
6. 到货地点： 昆明市云南省烟草烟叶公司昆明市
7. 加工单位： 云南省烟草烟叶公司
8. 备注： 省储购销/散叶/云系

**Figure S2.** Chart of documents to be identified

As shown in Table S1, traditional OCR methods (e.g., PaddleOCR) struggle to accurately recognize document images containing overlapping and misaligned characters. This limitation arises from the significant displacement of target regions and the presence of character overlap, leading to issues such

**Table S1.** Demonstration of recognition results using different models for FigureS2

| **text field** | **Corresponding Matching Text Fields** | **Paddle OCR** | **ChatGPT_4o** | **DeepSeek_R1** | **Qwen2.5-Max** | **Our method + Paddle ocr** |
|---|---|---|---|---|---|---|
| 产地 | 中国 | 产南省烟草公 | 云南省烟草公司文山州公司 | 未直接提及 | 云南省烟草公司文山州公司 | 中国 |
| 类型 | 烤烟 | （空） | 烤烟 | 批烟-原烟形态 | 烤烟 | 烤烟 |
| 形态 | 把烟-原烟 | （空） | 把烟-原烟 | （原烟形态） | 把烟-原烟形态 | 把烟-原烟 |
| 品种 | 其他 | （空） | （空） | 等级B1F | 其它 | 其他 |
| 生产年度 | 2022 | （空） | 2022 | 未直接提及 | 2022 | 2022 |
| 等级 | 上桔一 | BF1 | 上梗一B1F | B1F | B1F | 上桔一 |
| 代号 | BIF | 68000 | 680.0000 | 680.0000 | 680.0000 | BIF |
| 数量（担） | 680.0000 | 3199.00 | 3199.00 | 3199.00 | 3199.00 | 680.0000 |
| 单 价（元/担） | 3199.00 | （空） | 3199.00 | 680.0000 | 2175320.00 | 3199.00 |
| 金额（元） | 2175320.00 | （空） | 2175320.00 | 2,175,320.00 | （空） | 2175320.00 |
| 运输方式 | 直运 | （空） | （空） | （未填写） | （未填写具体方式） | 直运 |
| 交货时间 | 2023-01-03至2023-03-04 | 2交0货23时-0间1：-03至2023-03-04 | 2023-01-03至2023-03-04 | 2023-01-03至2023-03-04 | 2023-01-03至2023-03-04 | 2023-01-03至2023-03-04 |
| 结算方式 | 托收承付 | （空） | （空） | （未填写） | 承兑汇票 | 托收承付 |
| 包装标准与质量 | 麻袋国际 | 麻袋国标 | 麻袋国标 | 纸袋固标 | 麻袋国标 | 麻袋国际 |
| 发货地点 | 云南省文山壮族苗族自治州 | 收承付 | 云南省烟草烟叶公司昆明市 | 云南省昆明市烟草烟叶公司 | 云南省文山壮族苗族自治州 | 云南省文山壮族苗族自治州 |
| 到货地点 | 昆明市云南省烟草烟叶公司昆明市 | 省烟草烟叶公司明到市货 地 云 点南； | 省 储 购 销/散叶/云系 | 未提及 | 昆明市云南省烟草烟叶公司昆明市 | 昆明市云南省烟草烟叶公司昆明市 |
| 加工单位 | 云南省烟草烟叶公司 | 加工单重位南：省文山壮族苗族自治州 | 云南省文山壮族 苗 族 自 治州；云南省烟草烟叶公司 | （重复标注，未填写） | 云南省烟草烟叶公司 | 云南省烟草烟叶公司 |
| 备注 | 省 储 购 销/散叶/云系 | （空） | （空） | 各省烟草烟叶公司 | 省 储 购 销/散叶/云系 | 省 储 购 销/散 叶/云系 |

as missing fields, incorrect label associations, and misidentified content. In contrast, large language models (e.g., ChatGPT-4o, DeepSeek-R1, and Qwen2.5-Max) exhibit stronger inference capabilities and are able to recognize most characters correctly. However, their performance in accurately matching fields remains limited. To address these challenges, the method proposed in this study reconstructs and realigns overlapping and misaligned characters, thereby transforming complex visual patterns into a more structured and tractable text recognition task. As a result, it achieves superior performance in both character recognition and field-to-field matching under difficult document conditions.

## 3 DATASET PRODUCTION DETAILS

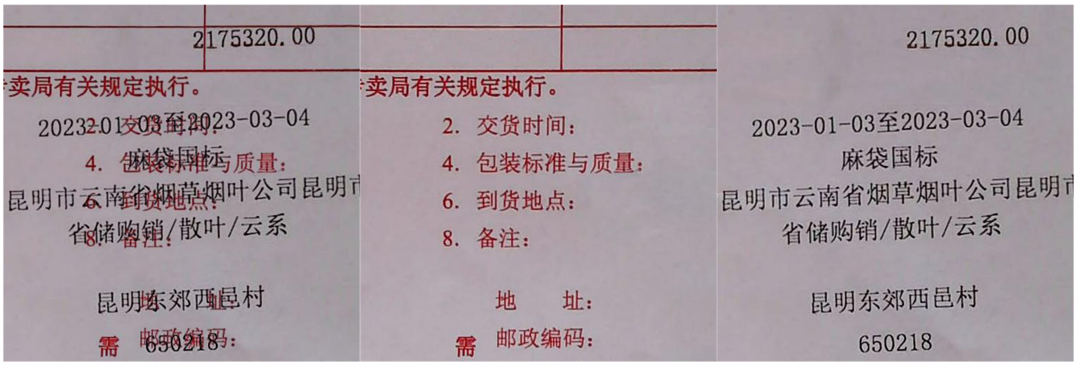


**Overlapping images of text** | **Background Label Image** | **Foreground Label Images**

**Figure S3.** Schematic diagram of the dataset (model input, foreground label, background label)

This study first constructed a document image dataset based on real-world scenarios. A total of 400 original document images were collected at a resolution of $1784 \times 2633$ pixels, encompassing two types of data: (1) document images with text overlap caused by double printing during actual office processes, and (2) images separately capturing foreground information (black text) and background information (red text) obtained from single-pass printing. It is important to note that the text regions in the foreground and background images are strictly aligned at the pixel level with those in the corresponding overlapped images, ensuring high spatial registration accuracy and annotation consistency across the dataset. These data accurately reflect the real-world issue of text overlap caused by repeated printing, providing a solid foundation for subsequent model development and training.

To further enhance the diversity and coverage of the dataset, additional synthetic overlapped document images were generated based on the foreground and background images obtained from single-pass printing. The synthesis process involved the following steps: first, separate images containing only black foreground text and only red background text were collected, ensuring that each image contained only a single text color. Then, random rotation and translation transformations were applied to the foreground and background images to simulate the misalignment and overlap commonly caused by printer shifts in real-world scenarios. The transformation parameters—including rotation angles and translation distances—were randomly sampled within predefined ranges, ensuring a diverse geometric distribution of the synthesized images. Finally, the transformed foreground and background images were fused to generate synthetic overlapped images containing both black and red text. By varying the rotation angle, translation offset, and blending transparency, the dataset captured a wide range of typical overlapping patterns, effectively representing the complexity found in real applications. Ultimately, 100 synthetic document images relevant to the tobacco

industry were created, expanding the total dataset to 500 images, along with 500 corresponding foreground and 500 background images.

To reduce the computational cost of model training and improve the precision of image detail restoration, all images were uniformly cropped into small patches of $128 \times 128$ pixels, while maintaining the spatial alignment among overlapped, foreground, and background images. This process yielded a total of 143,349 image patches as the initial dataset. Considering that some patches may contain completely overlapping foreground and background content (i.e., lacking valid overlapping information), we further computed the L1 difference between each overlapped image and its corresponding foreground and background images. A histogram of these differences was analyzed, and a threshold of 1.5 was used to filter out low-difference samples. After data cleaning, 127,017 valid patches were retained for each of the three image types (overlapped, foreground, and background).

For dataset partitioning, the images were split into training, validation, and test sets in an 8:1:1 ratio. Specifically, 114,679 patches were allocated to the training set, 12,701 to the validation set, and 12,701 to the test set for each image category.

The final dataset consists of the following three types of images:

**Overlapped images (model input):** Containing both black foreground and red background text, these serve as inputs for training the model to restore images under text-overlapping conditions.

**Foreground label images:** Containing only black text, these serve as supervision targets for foreground recovery, guiding the model in separating and reconstructing foreground information.

**Background label images:** Containing only red text, these serve as supervision targets for background recovery, assisting the model in extracting and restoring background information.

It is worth emphasizing that the three types of images are strictly aligned at the pixel level, ensuring annotation accuracy and sample consistency during supervised training.

### 3.1 Generalization to Large-size Document Images

To evaluate the scalability of the proposed TORNet on real-world industrial documents, we conducted additional experiments on images with larger spatial dimensions. Although the model was trained on relatively small patches of $128 \times 128$, we assessed its performance on $512 \times 512$inputs during inference. The results are summarized in Table S2.

**Table S2.** Performance of TORNet on different input image sizes.

| Image Size | PSNR (dB) | SSIM |
|---|---|---|
| $128 \times 128$ | 36.38 | 0.979 |
| $512 \times 512$ | 36.35 | 0.978 |

As shown, when the image size increases by a factor of 16, the model maintains stable performance with negligible degradation in both PSNR and SSIM. This demonstrates that TORNet possesses strong scale-robust feature extraction and context modeling capabilities.

To further support deployment on full-size industrial documents, we adopt a sliding-window strategy with either non-overlapping or slightly overlapping patches (e.g., $128 \times 128$or $512 \times 512$) during inference. Each

patch is processed independently, and the outputs are seamlessly fused to reconstruct the entire document image. This strategy mitigates memory limitations while preserving prediction consistency and spatial continuity, making the model suitable for practical industrial applications.

## 3.2 Practical Deployment in Industrial Systems

To evaluate the real-world applicability of the proposed TORNet framework, we integrated the image restoration and alignment model into a tobacco industry document digitization system, where it serves as the core back-end image processing module. The system is deployed on a local server and is designed to handle business documents that suffer from character overlap and misalignment caused by double printing.

During operation, document images are first captured by an image acquisition module (e.g., overhead scanners), followed by manual identification of character overlap regions. If overlapping is detected, the images are fed into the proposed model, which reconstructs clean foreground and background images. Subsequently, a key-field matching and alignment algorithm fuses the restored components into a standardized, clean document. The processed image is then passed to an OCR module for key information extraction. The extracted fields are matched with existing business records through a backend system built on the Odoo framework, which supports data comparison, storage, and audit management.

The full system includes front-end interaction, image acquisition, OCR calling, restoration and alignment, field comparison, and backend data handling, forming a comprehensive workflow with strong applicability and scalability. The system has been successfully deployed in a company. Due to the sensitivity of tobacco-related information, we are currently working on anonymizing the technical components. In the future, we plan to release a generic version of the system for broader industrial use.

The practical system interface and architecture are illustrated in the figures below:

The proposed method significantly improves OCR recognition accuracy in character-overlapping scenarios and shows promising potential for practical deployment in industrial document processing workflows.

## 3.3 Runtime Efficiency Evaluation

To evaluate the practical applicability of the proposed document restoration and alignment system, we assessed its end-to-end processing efficiency under varying input resolutions. All experiments were conducted using PyTorch 1.13 on a single NVIDIA GeForce RTX 4090 GPU with 24 GB of memory.

The complete pipeline comprises two primary stages: (1) document image restoration, and (2) key-field matching with alignment and fusion. We measured the average runtime (in seconds) for each stage using two representative input sizes ($128 \times 128$and $512 \times 512$), and the results are summarized in Table S3.

**Table S3.** Average runtime per sample (in seconds) under different input resolutions.

| Image Size | Restoration Time (s) | Alignment Time (s) | Total Time (s) |
|---|---|---|---|
| $128 \times 128$ | 0.13 | 0.012 | 0.14 |
| $512 \times 512$ | 1.18 | 0.038 | 1.22 |

The results demonstrate that our system achieves fast inference on low-resolution inputs, with a total average processing time of only 0.14 seconds per sample. For high-resolution inputs ($512 \times 512$), the processing time increases to approximately 1.22 seconds, which remains acceptable for practical

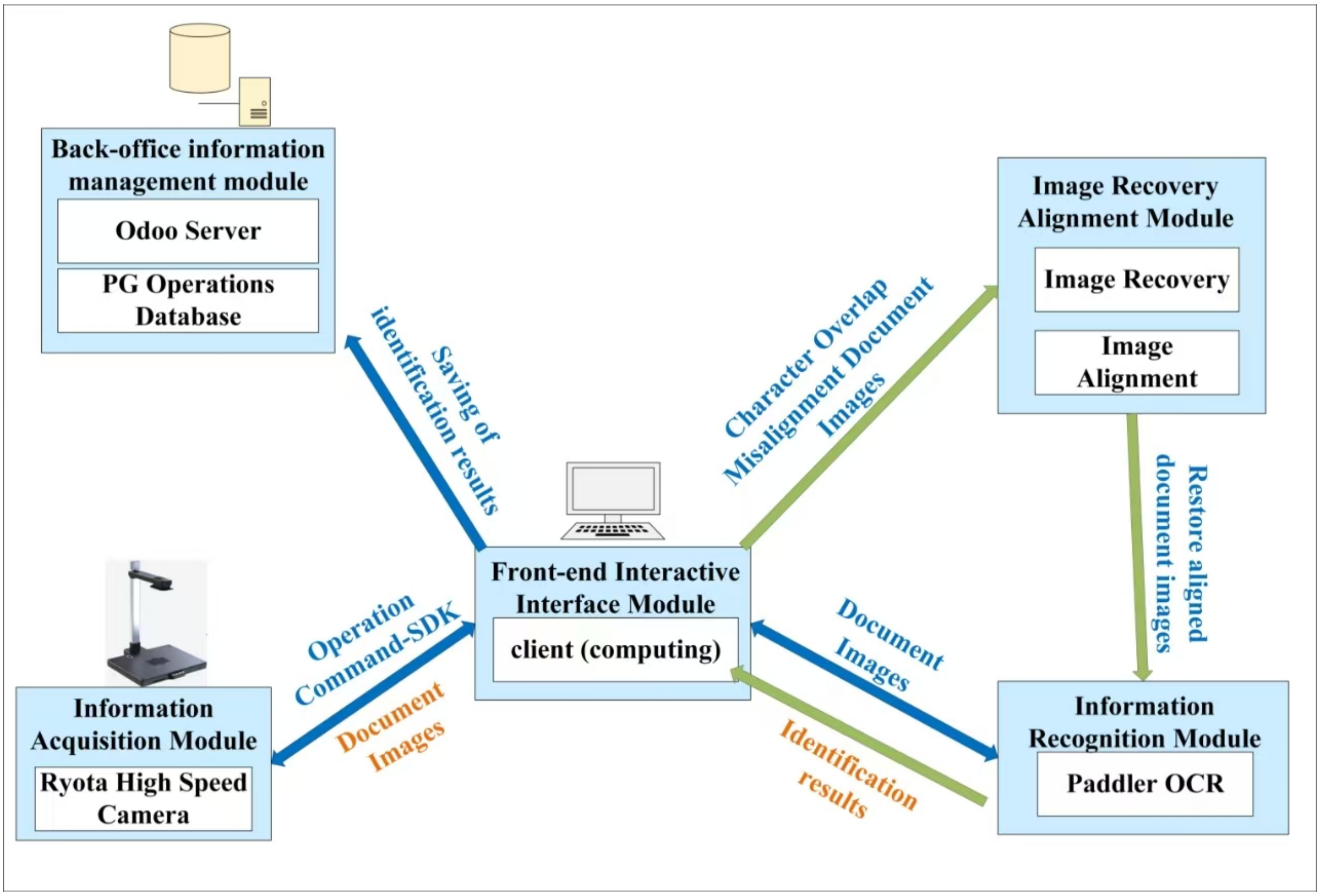


**Figure S4.** System architecture comprising five major modules: information management, front-end interface, image acquisition, OCR recognition, and image restoration-alignment.

deployment in industrial scenarios. These findings confirm that the proposed method effectively balances restoration quality and computational efficiency.

It is worth noting that actual runtime performance may vary depending on hardware specifications and input resolution. Therefore, system parameters can be flexibly adjusted to accommodate specific deployment requirements.

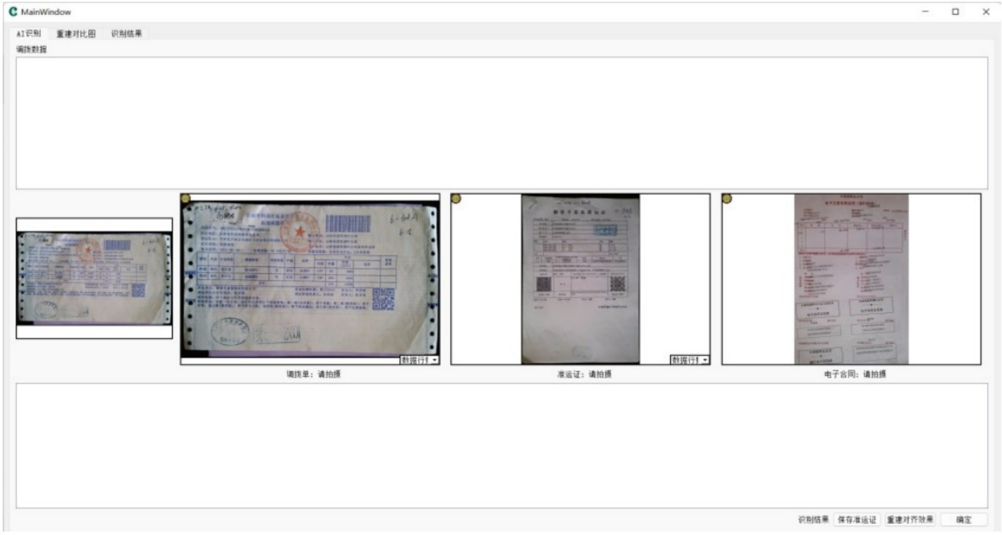


**Figure S5.** Front-end photo capture interface showing image data collected from the acquisition module.

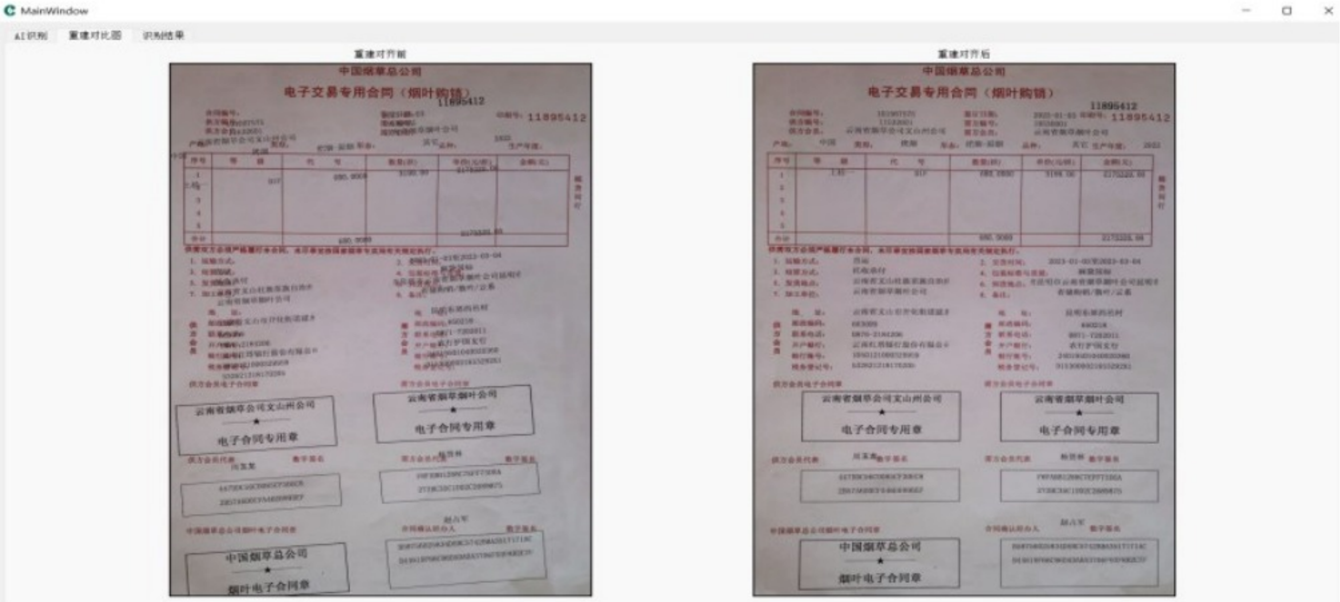


**Figure S6.** Visual comparison of a document before and after image restoration and alignment.

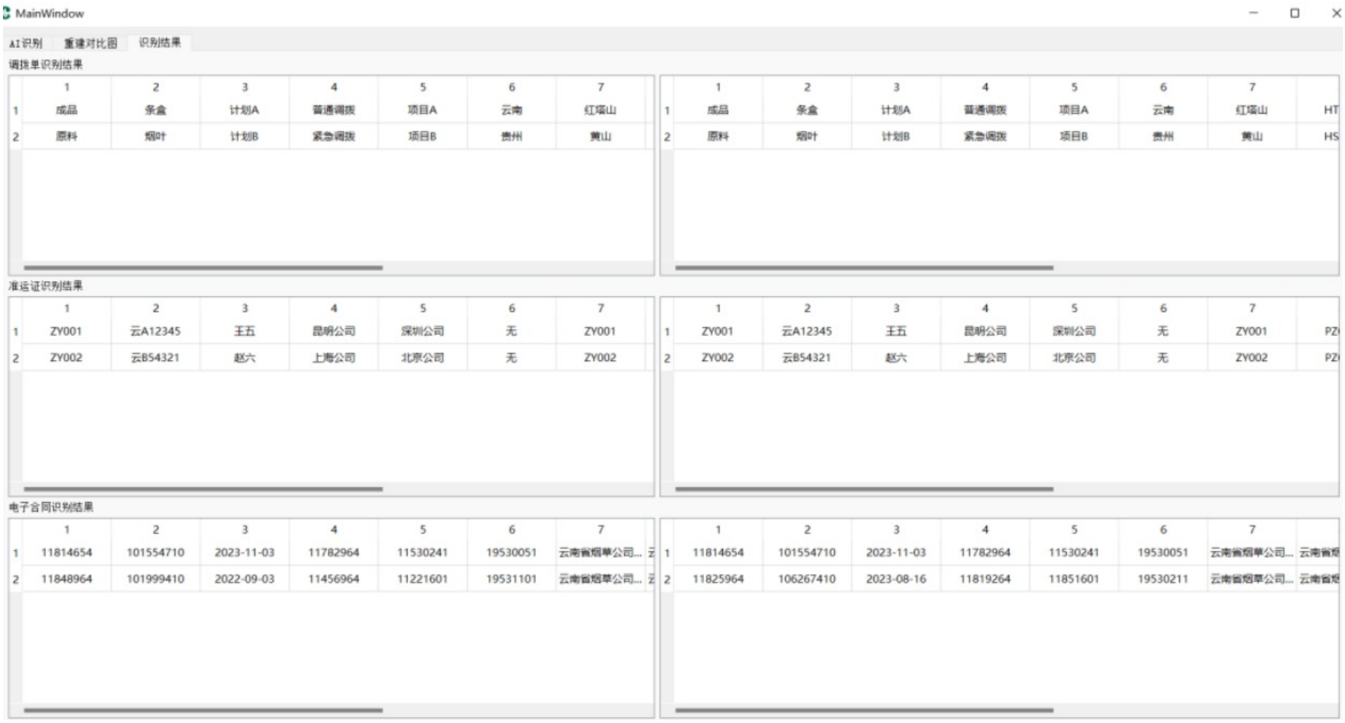


**Figure S7.** Recognition results returned by the OCR module for restored and original document images.

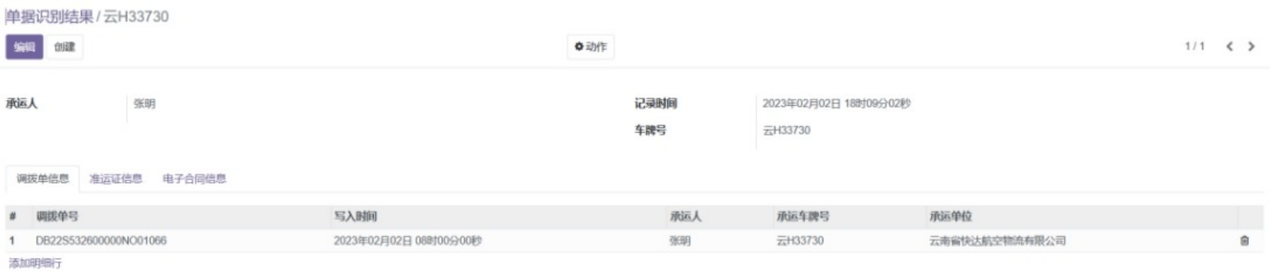


**Figure S8.** Backend information management interface displaying matched and stored field results.
