# Supplementary figures and images for: Image restoration and key field alignment for misaligned overlapping text in secondary printing document images
Source: Front Artif Intell. 2025 Sep 1;8:1616007. doi: 10.3389/frai.2025.1616007 (PMC12434626; doi:10.3389/frai.2025.1616007)

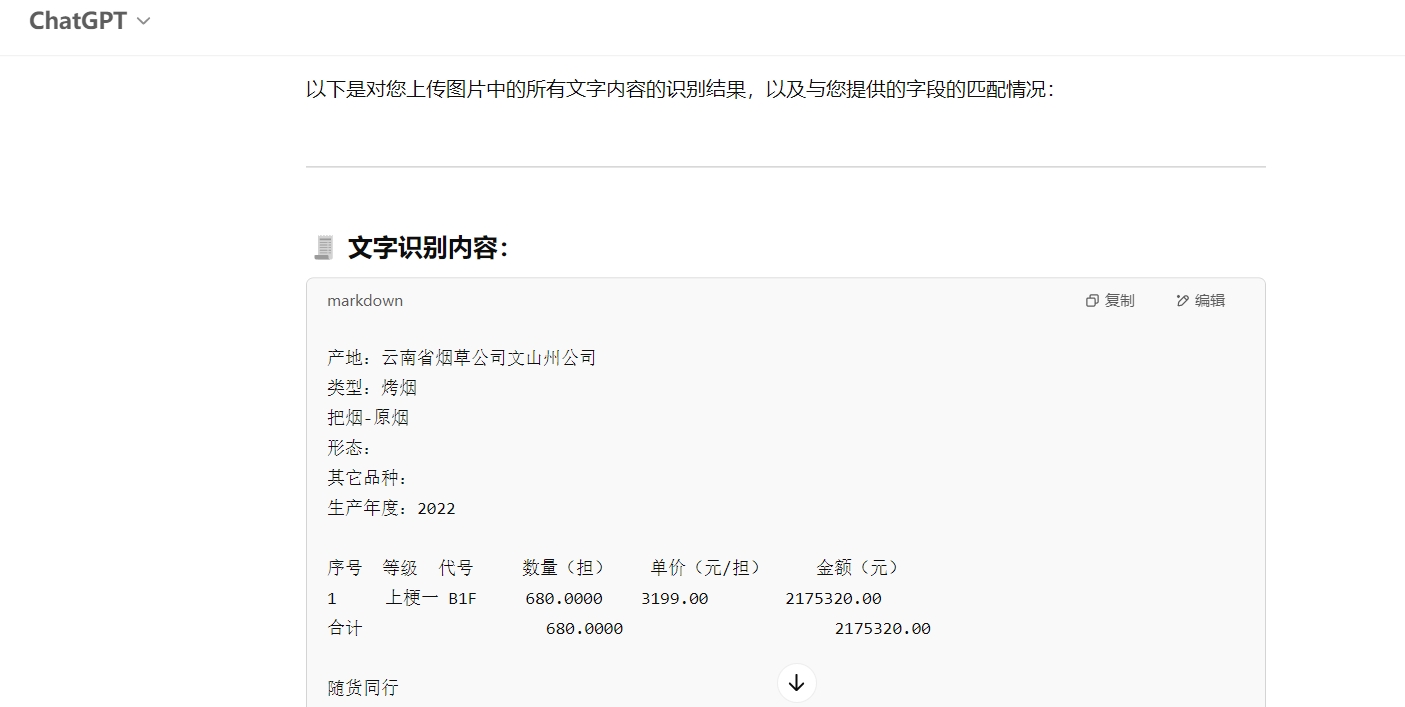

Supplement: Supplementary file 2 [file Image_1.png]

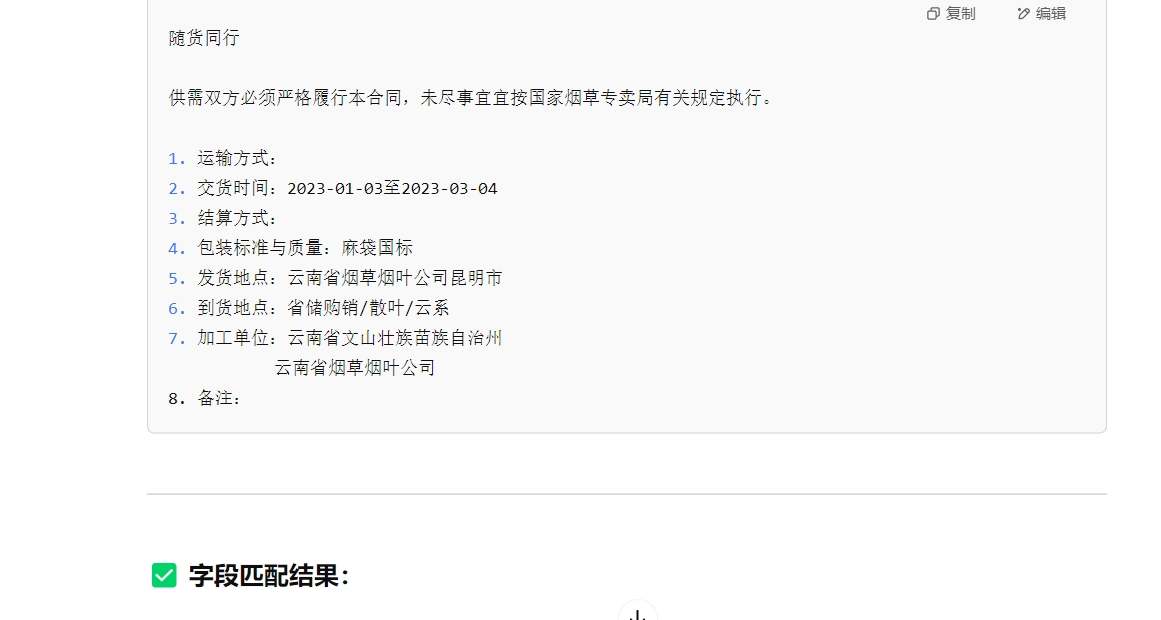

Supplement: Supplementary file 3 [file Image_2.png]

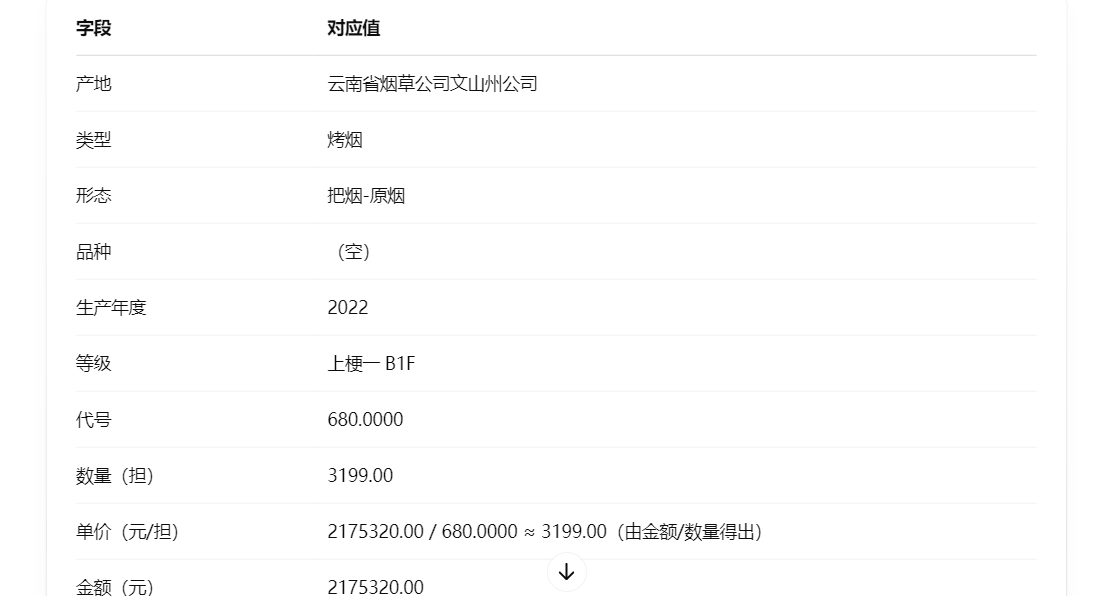

Supplement: Supplementary file 4 [file Image_3.png]

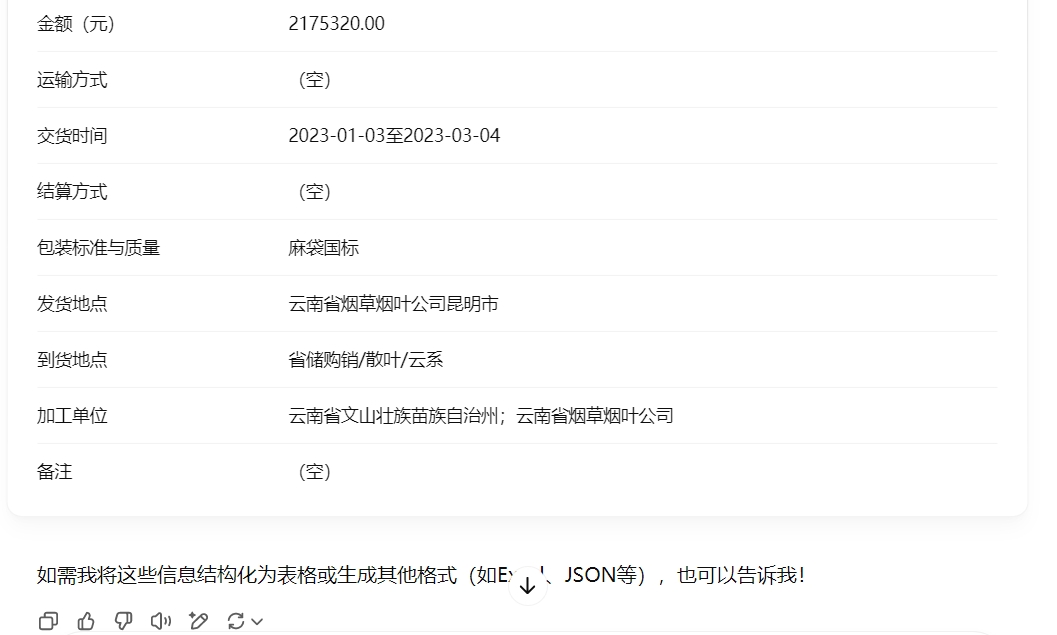

Supplement: Supplementary file 5 [file Image_4.png]

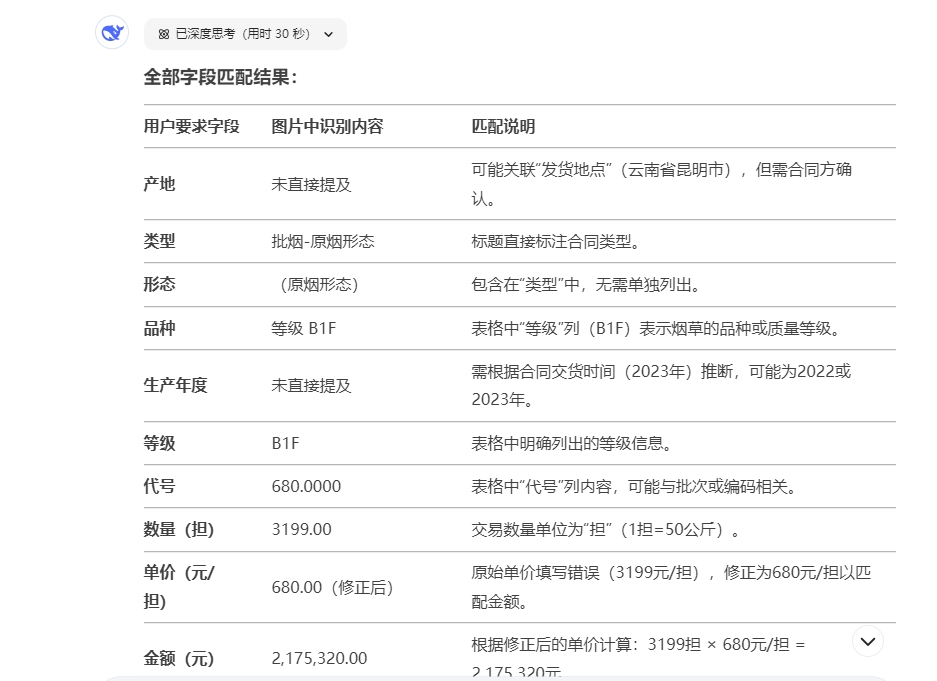

Supplement: Supplementary file 6 [file Image_5.png]

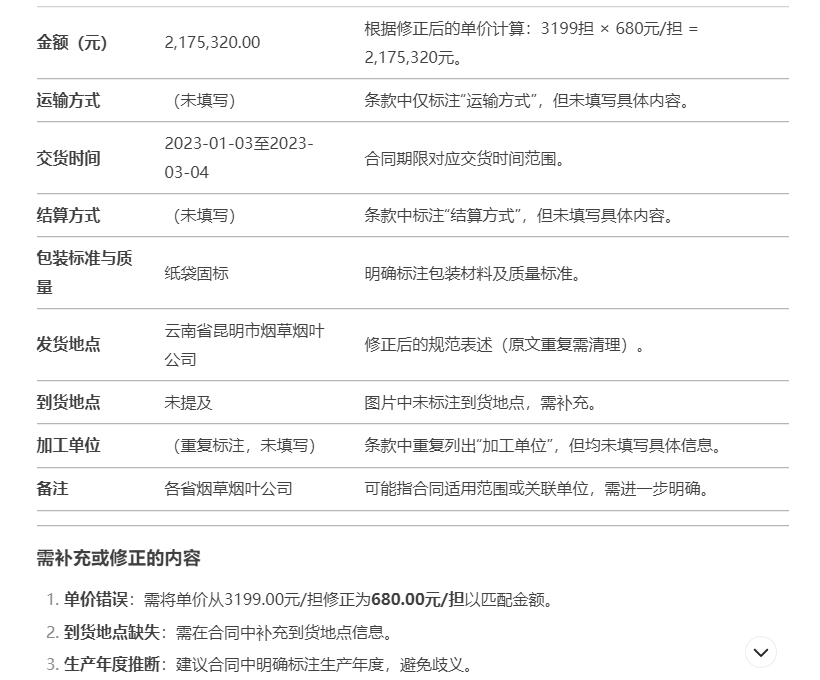

Supplement: Supplementary file 7 [file Image_6.png]

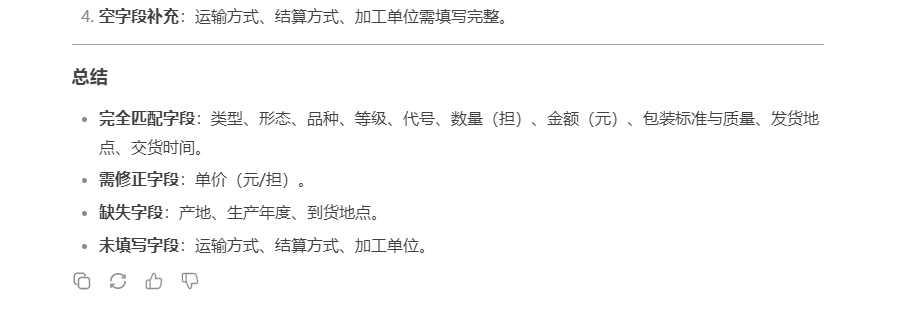

Supplement: Supplementary file 8 [file Image_7.png]

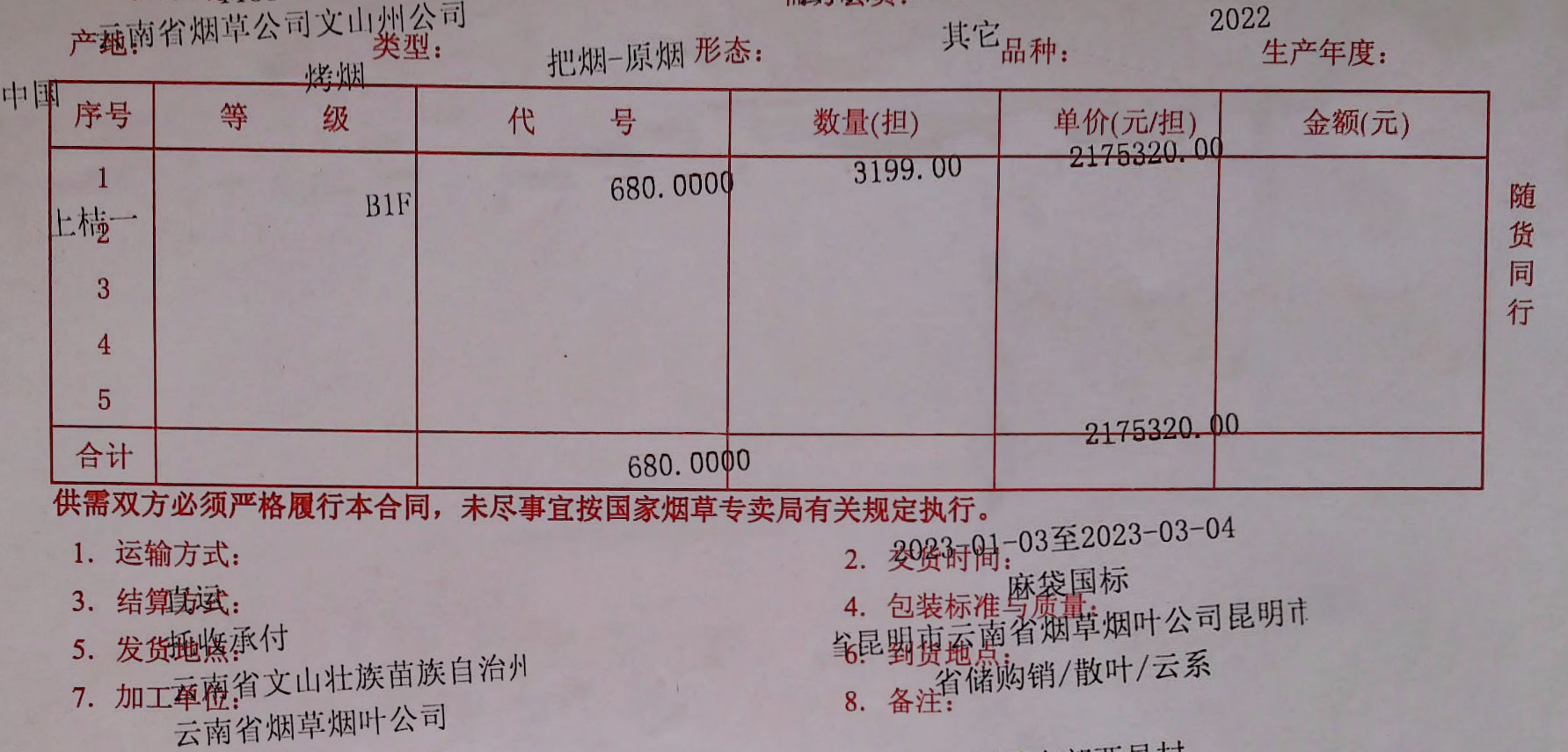

Supplement: Supplementary file 9 [file Image_8.png]

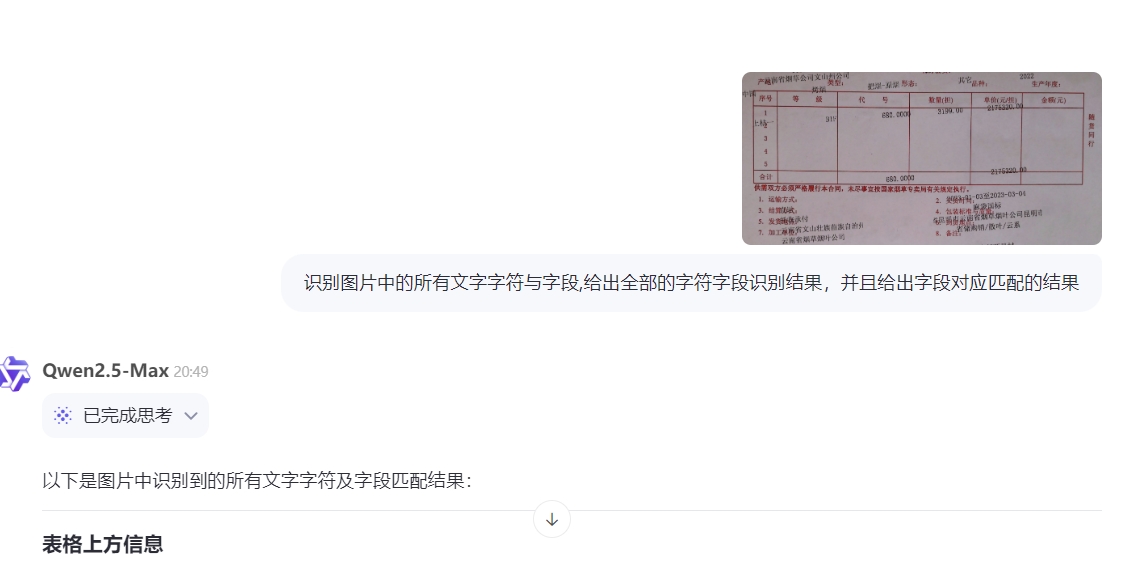

Supplement: Supplementary file 10 [file Image_9.png]

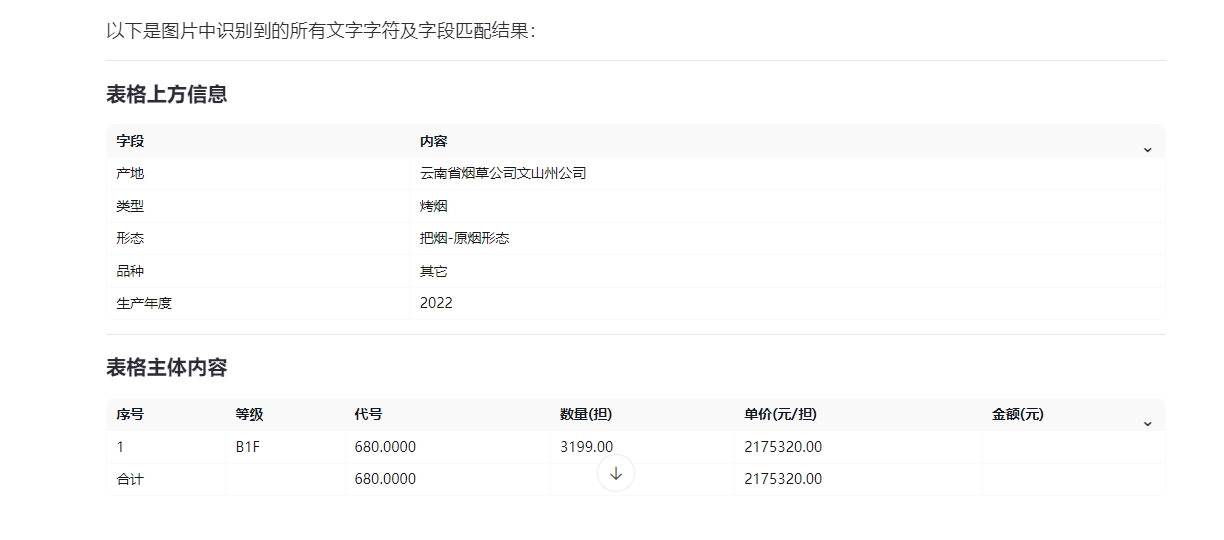

Supplement: Supplementary file 11 [file Image_10.png]

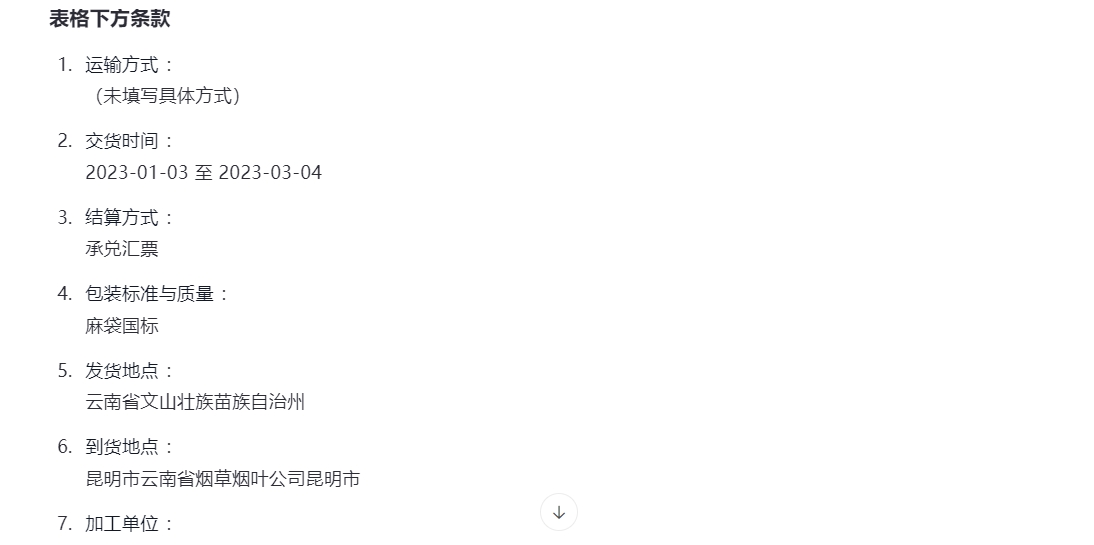

Supplement: Supplementary file 12 [file Image_11.png]

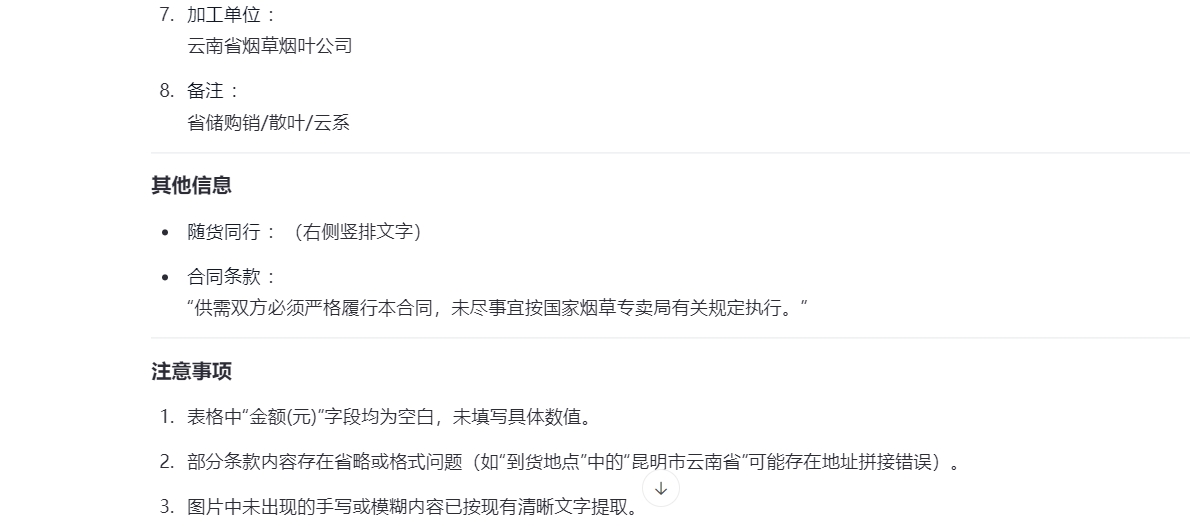

Supplement: Supplementary file 13 [file Image_12.png]
